# Temporal Evolution, Source Apportionment, and Health Risks of Atmospheric Halocarbons: A Case Study in the Central Yangtze River Delta Region

**DOI:** 10.3390/toxics13121085

**Published:** 2025-12-17

**Authors:** Yuchun Jiang, Anqi Zhang, Qiaoli Zou, Hanfei Zuo, Jinmei Ding, Lu Zhang, Lingling Jin, Da Xu, Yuwen Niu, Bingye Xu, Xiaoqian Li

**Affiliations:** 1 State Key Laboratory of Environmental Criteria and Risk Assessment, Chinese Research Academy of Environmental Sciences, Beijing 100012, China; 2 Ecological and Environmental Monitoring Center of Zhejiang Province, Hangzhou 310012, China; 3 College of Environmental and Resource Sciences, Zhejiang Provincial Key Laboratory of Organic Pollution Process and Control, Zhejiang University, Hangzhou 310058, China; 4 Zhejiang Institute of Meteorological Sciences, Hangzhou 310057, China

**Keywords:** halocarbons, long-term trends, source apportionment, regional transport, health risk assessment

## Abstract

Recently, the environmental impact of halocarbons has become increasingly concerning, particularly due to the growing influence of non-regulated halocarbons on stratospheric ozone depletion and their adverse health effects in the troposphere. Previous model studies have highlighted the importance of halocarbon emissions from the YRD. However, only several reports have discussed the long-term pollution characteristics and health risks of halocarbons in the YRD based on observational data. The continuous observation of halocarbons was conducted in the central part of the YRD (Shanxi site) from 2018 to 2023. The result showed that rise in halocarbon levels was primarily driven by alkyl halides, including dichloromethane (1.194 ppb to 1.831 ppb), chloromethane (0.205 ppb to 1.121 ppb), 1,2-dichloroethane (0.399 ppb to 0.772 ppb), and chloroform (0.082 ppb to 0.300 ppb). The PMF and CBPF analysis revealed that pharmaceutical manufacturing (37.0% to 60.2%), chemical raw material manufacturing (8.0% to 19.9%), solvent use in machinery manufacturing (12.4% to 24.7%), solvent use in electronic industry, and background sources were the main sources of halocarbons at the Shanxi site. Among them, the contributions of chemical raw material manufacturing, as well as of solvent use in machinery manufacturing and electronic industry, are increasing. These aspects are all dominated by local emissions. Furthermore, the carcinogenic risks of chloroform and 1,2-dichloroethane, which rank first in this regard, are increasing. Also, attention should be paid to solvent use in the electronic industry and the background. The probabilities of these activities coming with health risks that exceed the acceptable levels are 94.8% and 94.9%. This study enriches the regional observation data in the YRD region, offering valuable insights into halocarbon pollution control measures for policy development.

## 1. Introduction

Halocarbons are important components of volatile organic compounds (VOCs). They are widely used in industrial manufacturing as blowing agents, refrigerants, solvents, and fire suppressants. However, due to their ozone-depleting potential in the stratosphere and contribution to the greenhouse effect, they are regulated under the Montreal Protocol and its amendments [1]. As of 2022, some regulated halocarbons (i.e., CFC-11 [2], carbon tetrachloride [3] and Halon-1211 [4] and others) have shown a declining trend.

In contrast, the global emissions of some non-regulated halocarbons, such as dichloromethane and chloroform, are increasing. Studies suggest that global emissions of dichloromethane rose from 637 Gg/year in 2006 to 1171 Gg/year in 2017 [5], with reported emissions reaching 1038 Gg/year in 2019 [6]. The emission of chloroform increased steadily until 2017 (2017: 345 Gg/year) and remained stable (2019: 339 Gg/year) [3]. Although these are historically considered to have a minor impact on stratospheric ozone due to their relatively short atmospheric lifetimes (<6 months), recent aircraft observations over tropical regions have revealed significantly elevated concentrations of non-regulated halocarbons in the tropopause [7] and the lower stratosphere [8]. Moreover, modeling studies indicated the presence of stratospheric chlorine [9] and bromine [10]. These findings highlight the growing contribution of non-regulated halocarbons to stratospheric chlorine loading, which adds further uncertainty in predicting the recovery timing of the ozone layer. In addition, non-regulated halocarbons are regarded as hazardous air pollutants that exert significant adverse health effects [11,12,13] and are prohibited or restricted in accordance with relevant regulations [14,15].

Emissions of non-regulated halocarbons from Asia, particularly eastern China, have been the primary drivers of global emission trends [6,16,17,18,19]. Claxton et al. [19] reported that Asia’s share of global dichloromethane emissions increased from 68% to 89% between 2006 and 2017, while An et al. [6] identified the North China Plain and the Yangtze River Delta (YRD) as the main contributors. Fang et al. [16] focused on chloroform emissions from eastern China between 2007 and 2015, and An et al. [17] used domestic observational data to estimate nationwide chloroform emissions from 2010 to 2020. Both studies found that the trends and magnitudes of China’s emissions during their respective study periods closely matched global changes.

However, observations in eastern China have primarily been concentrated in the North China Plain region [18,20,21,22] and the Pearl River Delta [23,24,25]. In contrast, research on near-surface halocarbon concentrations in the YRD remains limited. Previous studies in the YRD region have mostly focused on regulated substances [26,27] and the short-term variation [28,29,30]. Jiang et al. [31] previously investigated temporal trends in halocarbon concentrations in the region, identifying a continuing increase. However, these studies have not addressed the long-term source evolution patterns of halocarbon emissions. Moreover, most health risk assessments are deterministic [32,33,34], in which each exposure parameter input into the model is represented by a single value. This approach may either overestimate or underestimate the actual risk [35].

In this study, halocarbons in the central YRD region (Shanxi site) were investigated using an online VOC monitoring system. Continuous observations were conducted from 2018 to 2023 to characterize the pollution features of halocarbons. Based on these data, major anthropogenic sources were identified using the positive matrix factorization (PMF) model, and their potential emission regions were further determined through the conditional bivariate probability function (CBPF) method. Following the exposure assessment and uncertainty analysis recommended by the U.S. EPA, the health risks associated with halocarbon species and their sources in this region were evaluated.

## 2. Materials and Methods

### 2.1. Study Area and Monitoring Methods

**Figure 1:**
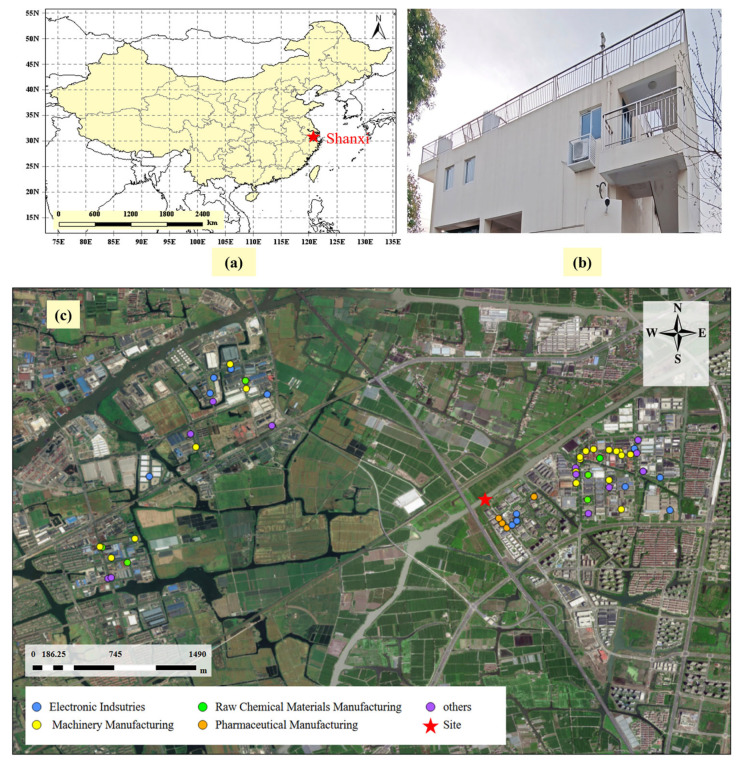
Location of Shanxi site (**a**). Photo of sampling site (**b**). Specific industries surrounding the monitoring site (**c**).

As shown in Figure 1, the monitoring site selected for this study (30°49′29″ N, 120°52′6″ E) was the Shanxi site (No. 330400102), which is part of the Zhejiang Atmospheric Composite Pollution Auto-Monitoring Net. The Shanxi site is surrounded by major industrial urban clusters of the YRD, such as Shanghai, Hangzhou, and Suzhou. Within a 3 km radius of the monitoring site, the area to the east consists of a mixed zone of industrial parks and residential areas, where the industrial parks mainly focus on traditional machinery manufacturing, electronic information products, chemical raw materials, and medical products. Extensive farmland and woodland are scattered to the north and south of the site, while approximately 5 km to the northwest lies another industrial park encompassing electronic manufacturing, machinery manufacturing, and plastic production.

An online monitoring instrument (TH-PKU 300B GC-MS/FID (Wuhan Tianhong Environmental Protection Industry Co., Ltd., Wuhan, China) [36]) with a time resolution of 1 h was employed in this study to observe the concentration of halocarbon. This equipment measures ambient halocarbons through four main stages: preparation, sampling and pre-concentration, GC analysis, and system heating back-flush. Ambient air samples first pass through a cold trap that removes impurities such as O_3_, H_2_O, and CO_2_. Subsequently, the sample is introduced into a low-temperature (−150 °C) enrichment trap. After pre-concentration, halocarbons are heated to the gaseous phase and analyzed by a chromatographic column coupled with a mass spectrometer. More details of this equipment can be found in Wang et al. [37]. The method detection limits (MDLs) for halocarbons were calculated following the EPA TO-15 guideline [38] and are summarized in Table S1. This continuous observation was conducted between 1 January 2018, and 31 December 2023. During the study period, the numbers of data points we obtained from each year were 6863, 7618, 7268, 7625, 7091, and 7168, respectively, with an overall data validity rate of 86.5%. Furthermore, we utilized the Lufft WS500-UMB [39] (Hach Water Quality Analytical Instrument (Shanghai) Co., Ltd., Shanghai, China) intelligent meteorological sensor to monitor various meteorological parameters. The meteorological parameters are presented in Table S2.

### 2.2. Positive Matrix Factorization Model

The PMF 5.0 model [40] was used to analyze the major sources and relative contributions at the Shanxi site. For a detailed description of the model, please refer to previous studies [41]. A brief introduction to the fundamental principle of the PMF model is provided below:

(1) $$X_{ij}=\sum_{k=1}^{p}g_{ik}f_{kj}+e_{ij}$$

In Equation (1), *x_ij_* represents the concentration of the *j*th VOC species measured in the *i*th sample; *g_ik_* denotes the contribution of the *k*th factor to the *i*th sample; *f_kj_* is the abundance of the *j*th species in the *k*th factor; *e_ij_* represents the residual for the *j*th species in the *i*th sample; and *p* is the number of sources. To reduce the rotational ambiguity of factors, the PMF model applies non-negativity constraints to parameters, minimizing the objective function *Q*, as shown in Equation (2):

(2) $$Q=\sum_{i=1}^{n}\sum_{j=1}^{m}{\left[\frac{e_{ij}}{u_{ij}}\right]}^{2}$$

where *u*th is the uncertainty of the *j*th halocarbon species in the *i*th sample. The uncertainties were calculated with Equation (3):

(3) $$u_{ij}=\left\{\begin{array}{cc} MDL\times \frac{5}{6} & x_{ij}\leq MDL\\ \sqrt{{\left(\frac{1}{2}\times MDL\right)}^{2}+{\left(EF\times x_{ij}\right)}^{2}} & x_{ij}>MDL \end{array}\right.$$

EF stands for error factor, which depends on the precision of the monitoring instruments. Based on the species selection criteria reported in previous studies [13], 20 halocarbon species were input into the model. Additionally, acetone and 1,3-butadiene were introduced as typical tracers. Acetone is a representative compound in industrial production [42] and 1,3-butadiene is commonly used as an indicator of synthetic manufacturing sectors such as rubber and plastics [43].

### 2.3. Conditional Bivariate Probability Function

By using a conditional bivariate polar plot model, the spatial distribution of pollutants can be visualized. Previous studies [44,45] have combined PMF source apportionment results with this approach to illustrate the location information of different emission sources. It can be defined as follows:

(4) $$CBPF_{\Delta \theta ,\Delta u}=\frac{m_{\Delta \theta ,\Delta u}}{n_{\Delta \theta ,\Delta u}}$$

In Equation (4), $m_{\Delta \theta ,\Delta u}$ represents the number of samples within the wind direction sector Δθ at a given wind speed Δu that exceed a certain threshold x, while $n_{\Delta \theta ,\Delta u}$ denotes the total number of samples within that wind speed and wind direction sector. In this study, the threshold is set at the 95th percentile of each source’s contribution, and the direction corresponding to the high-value points is used to determine the direction of each pollution source [46,47]. A detailed introduction to CBPF can be found in Carslaw et al. [48].

### 2.4. Health Risk Assessment

To assess the health risks posed by halocarbons in the atmospheric environment, this study first employed the point evaluation method recommended by the U.S. EPA [49]. The carcinogenic and non-carcinogenic risks resulting from inhalation exposure can be calculated using the following formulas:

(5) R=IUR×EC

(6) EC=(CA×ET×EF×ED)/AT

(7) HQ=EC/RfC

(8) $$HI=\sum HQ_{i}$$

Equations (5) and (6) are used to assess carcinogenic risk, where *R* represents the cancer risk, *IUR* is the inhalation unit risk (m^3^/μg), and *EC* is the exposure concentration (μg/m^3^). *CA* refers to the concentration of the pollutant in air (μg/m^3^), *ET* is the exposure time (hours/day), *EF* is the exposure frequency (days/year), *ED* is the exposure duration over a lifetime (years), and *AT* is the average time (hours). Equations (7) and (8) are employed to evaluate non-carcinogenic risk by calculating the dimensionless hazard quotient (*HQ*). *RfC* denotes the reference concentration (μg/m^3^), which represents the inhalation exposure level at or below which no appreciable health effects are expected to occur over a lifetime. The *RfC* values and the *IUR* values for each halocarbon are listed in Table S3.

This study also employed the Monte Carlo simulation method to quantify the probabilistic distribution of health risks associated with individual compounds and source categories [50]. The simulations were conducted using Crystal Ball 11.1 software, with the number of iterations set to 10,000. The probability distributions of parameters used in the model are presented in Tables S4 and S5, in which the exposure time, exposure duration, and average time refer to the standards outlined in the Chinese Exposure Factors Handbook [51].

## 3. Result and Discussion

### 3.1. Characteristics of Halocarbon Concentrations

This study conducted continuous observations at the Shanxi site from 2018 to 2023, including 16 alkyl halides, 7 alkenyl halides, and 5 aryl halides. The 6-year surface observation campaign at the Shanxi site shows the distinctive yearly change, with a range from 3.013 ppb to 4.774 ppb (Figure S1). An overall increasing trend in concentrations was observed except for the period between 2020 and 2022. During this time, the outbreak of COVID-19 in China and the governmental control measures significantly impacted regional production activities, causing a sharp decline in emissions from some halocarbons sources [52]. Notably, the concentration of aryl halides increased from 0.223 ppb in 2019 to 0.240 ppb in 2020, with their percentage contribution rising by 1.8% (Figure S1). This indicates that certain sources of halocarbon emissions remained unaffected by restrictions.

To clarify the influence of human activities on halocarbon concentration, the monthly variation was analyzed. As shown in Figure S2, halocarbon concentrations generally decreased in February, which could be related to the official Chinese Spring Festival holiday period, during which surrounding industrial activities typically slow down. Between 2018 and 2022, the concentration dropped from January to February and ranged from 0.092 ppb to 1.889 ppb. This pattern was particularly pronounced in 2020, when the total concentration dropped to 0.583 ppb. This can be attributed to the policy of extending the Spring Festival holiday [53] and a tiered return-to-work policy implemented by local enterprises [54]. Concentrations of halocarbons began to rise from March to May. A subsequent decrease was observed during June, July, and August, with levels being affected by the enhancement of photochemical reactions and the increase in boundary layer heights during the summer. This seasonal pattern was evident from 2018 to 2021. However, the downward trend was less apparent in 2022 and 2023. According to reports from the National Climate Center, temperatures in parts of the YRD during June 2022 and 2023 were 2–4 °C higher than the historical average [55]. As the evaporation rates and emission strengths [56] of VOCs are temperature-dependent, it is speculated that the frequent extreme heat events in 2022 and 2023 contributed to the elevated concentrations during this period. Halocarbon concentrations began to rise again after September, reaching relatively high levels in December each year. From 2019 to 2023, December concentrations ranged from 2.959 ppb to 6.65 ppb. This was associated with suppressed boundary layer and poor dispersion conditions during the winter season. These results further support previous findings that VOC concentrations in the YRD tend to be higher in winter than that in summer [57,58].

The concentrations and average contributions of 28 halocarbons are displayed in Table S6. Given that some halocarbons present greater threats to public health than others, 13 priority halocarbons were selected based on the Urban Air Toxics List [15]. To emphasize the features of Shanxi site, comparative analysis was conducted with simultaneous observations from other YRD regions.

As shown in Table 1, dichloromethane and chloromethane were the most abundant halocarbons in the study area. Both exhibited an overall increasing trend, with a concentration decrease observed in 2020. Their concentration ranges were 1.194–1.831 ppb and 0.201–1.121 ppb, respectively. Dichloromethane is predominantly influenced by anthropogenic sources, including industrial solvent usage [29], foam manufacturing, and emissions from HFC production [6,7]. Although chloromethane is globally considered to originate primarily from natural sources [3] and is recognized as a tracer for biomass burning [59], studies have shown that the increasing trends in eastern China are strongly affected by anthropogenic activities [60]. These sources include the production of methyl chloride, solvent use in plastic and rubber industries, HCFC/HFC manufacturing, and coal combustion in industrial processes [61,62,63]. In this study, chloromethane levels were comparable to those observed at the Shangdianzi site (0.810 ppb) [22] and in the industrial area of Handan (1.290 ppb) [64], but significantly higher than those in the clean air mass at the Shangdianzi site (0.534 ppb) [22]. This suggests that the continued increase in chloromethane levels in this study may be led by intensified industrial usage. Additionally, dichloromethane and chloromethane concentrations at the two Zhejiang sites (Hangzhou and Shanxi) were higher than those in Nanjing, likely due to differences in dominant industrial sectors between two regions.

The concentration of 1,2-dichloroethane exhibited a fluctuating increase over the study period, rising from 0.632 ppb to 0.772 ppb, whereas 1,2-dichloropropane showed a declining trend, decreasing from 0.217 ppb to 0.121 ppb. Compared with Nanjing, concentrations of both compounds were relatively lower in the Zhejiang sites. These substances are commonly used as solvents in industrial processes such as machinery manufacturing [65,66]. The concentrations of tetrachloroethylene and vinyl chloride remained relatively stable, ranging from 0.037 to 0.041 ppb and 0.020 to 0.058 ppb, respectively, with no significant differences observed across the YRD region. Tetrachloroethylene and vinyl chloride are representative compounds used in the dry-cleaning industry [67] and plastic manufacturing sectors [68], respectively.

In contrast, the levels of chloroform, trichloroethylene, and 1,1-dichloroethane at the Shanxi site have shown continuous upward trends. The concentration ranges for chloroform and 1,1-dichloroethane were 0.082 ppb–0.300 ppb and 0.005 ppb–0.417 ppb, respectively, representing approximately 10-fold and 2-fold increases in 2020–2023 compared to 2018–2019. Similarly, the concentration of trichloroethylene increased from 0.032 ppb to 0.070 ppb. Trichloroethylene is commonly used as a cleaning agent in dry cleaning and electronics manufacturing [66,69,70,71], while 1,1-dichloroethane is a good solvent used as dry-cleaning agent for electronic components [72] and fabric manufacturing [73]. The increases in these substances are related to the electronic manufacturing nearby Shanxi site, which is a key emerging industry in Jiashan County [74].

Additionally, the presence of some substances is relatively low and virtually unchanged during the studying period, such as 1,2-dibromoethane, trans-1,3-dichloropropene, and cis-1,3-dichloropropene. Their concentrations in Nanjing are considerably higher than those at the Hangzhou and Shanxi sites in Zhejiang. The mixture of 1,3-dichloropropene is widely used in agriculture as a pre-plant–soil fumigant [75] and 1,2-dibromoethane was once used as a pesticide ingredient but has been banned under relevant Chinese regulations [76]. These substances can pose great health risk, emphasizing the importance of potential transport from other regions of the YRD region.

### 3.2. Emission Source Profiles and Potential Sources Regions of Halocarbons

#### 3.2.1. Emission Source Profiles at Shanxi Site

**Figure 2:**
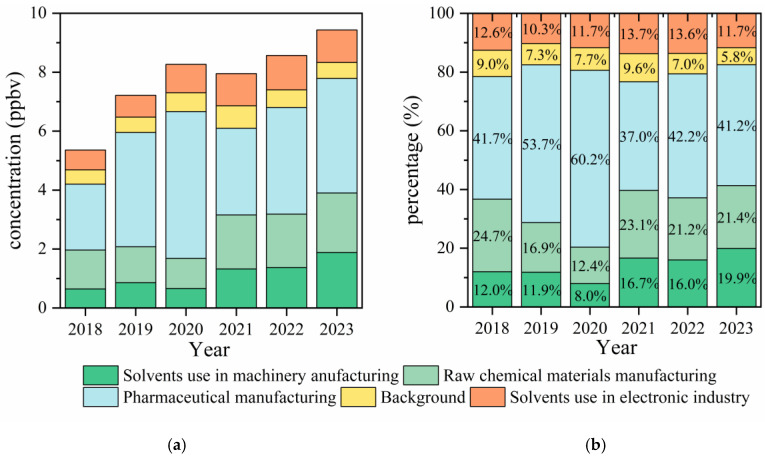
The concentrations (**a**) and contributions (**b**) of halocarbon sources derived from PMF during the period of 2018–2023.

The PMF model has identified the major emission sources of halocarbons at the Shanxi site from 2018 to 2023, including solvent use in machinery manufacturing, the manufacturing of chemical raw materials, pharmaceutical manufacturing, background, and solvent use in electronic industry. The identification process for each factor is descripted in Text S1 and the yearly source profiles are shown in Figure S3. The validation of 5-factor solutions can be found in Text S2, Text S3, Tables S7 and S8. Figure 2 presents the percentage contributions of the five source factors to halocarbon concentrations at the Shanxi site. Pharmaceutical manufacturing (37.0–60.2%) and the production of chemical raw materials (12.4–24.7%) were the dominant sources. Solvent use in the electronic industry and machinery manufacturing was the secondary contributor, with contributions ranging between 10.3 and 13.7% and between 8.0 and 19.9%, respectively.

From 2018 to 2023, the contribution of pharmaceutical manufacturing showed a continuous increase, with its contribution to ambient halocarbon levels reaching 60.2% in 2020. The significant rise in 2020 was largely driven by the policies adopted during COVID-19 pandemic, aiming to improve the local pharmaceutical industries [77]. Additionally, the large-scale use of disinfectants reported in China after the outbreak [78,79] may have contributed to increased levels of chlorinated disinfection by-products [80,81], ultimately leading to the rise in by-products observed in relation to the pharmaceutical manufacturing source.

The contributions of chemical raw material manufacturing and machinery manufacturing both increased, except for the year 2020. In 2020, they decreased by 4% and 5% compared to 2019; this was correlated with the decline in production [81]. In addition, some studies reported a decrease in VOC emissions from the automobile manufacturing and maintenance industry in China from 2019 to 2020 [82], which is generally consistent with the decline observed in this study.

From 2018 to 2023, the concentration of solvents used in the electronics industry increased from 0.67 ppb to 1.16 ppb, while its contribution rate remained approximately 14.0%. This rise is in agreement with the rapid growth of Zhejiang’s electronics information industry in 2020, driven by IC manufacturing in Shanxi county [83]. The value-added output of Zhejiang’s above-scale electronic information manufacturing industry increased by 16.8% that year [84].

The contribution of background sources fluctuated within the range of 6.8% to 9.0%. Although the levels of representative substances in background sources, such as CFC-11 and carbon tetrachloride, continuously declined, the contributions of dichloromethane and 1,2-dichloroethane in this source increased. This could be due to the enhanced fugitive emissions at the studying site [85] or the influence of halocarbon emission sources not considered in this study, such as coal combustion [64], biomass burning [29], or the steel manufacturing industry [86].

#### 3.2.2. Potential Sources Regions of Halocarbons at Shanxi Site

**Figure 3:**
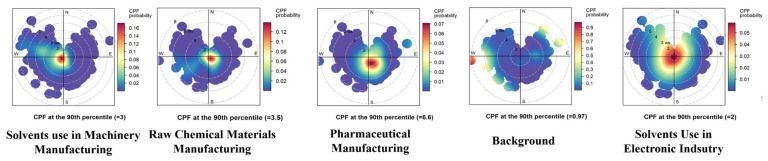
CBPF for the sources of halocarbons at the Shanxi site during the period of 2018–2023.

The CBPF analysis shows that the emissions from solvent use in machinery manufacturing, chemical raw materials, and solvent use in the electronic industry are predominantly local (Figure 3). The pharmaceutical manufacturing source is mainly associated with local and southeastward emissions, corresponding to the potential geographic location of medical production enterprises which are depicted in Figure 1. Representative substances of the background source are regulated compounds, such as CFC-11, CFC-113, and carbon tetrachloride. High values are detected for this source in the east, with speeds exceeding 5 m/s. Jiaxing city is located on the border of Zhejiang province, adjacent to a metropolitan area with a developed industrial base. The transportation of regulated halocarbons from these areas causes the elevation of background concentrations at the Shanxi site. Solvent use in the electronic industry was observed in all directions throughout the study period, reflecting its importance as a key emerging industry in Shanxi County.

### 3.3. Health Risk Assessment at Shanxi Site

#### 3.3.1. The Health Risk of Species

This study assessed the non-carcinogenic health risks of 19 halocarbons. As shown in Figure S4, the hazard index (HI) for non-carcinogenic risk ranged from 4.89 × 10^−2^ to 9.32 × 10^−2^, with all species remaining below the threshold set by the U.S. EPA. According to Figure S5, 1,2-dichloropropane and trichloroethylene were the compounds with the highest non-carcinogenic risk levels ranging, from 1.80 × 10^−2^ to 4.20 × 10^−2^ and from 1.18 × 10^−2^ to 4.56 × 10^−2^, respectively.

**Figure 4:**
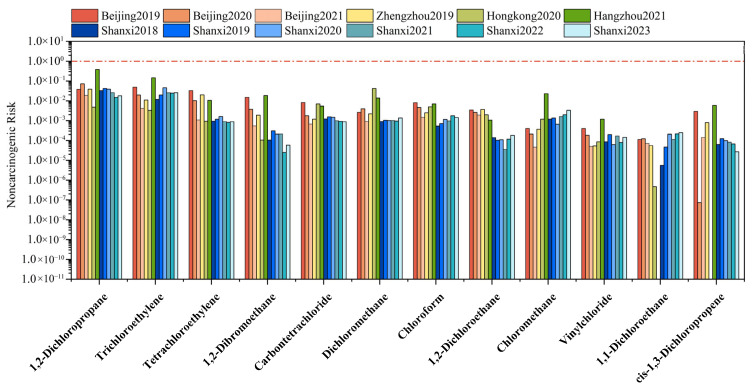
Comparison of the non-carcinogenic risk of selected halocarbons between the Shanxi site and other cities. The red line denotes the threshold of 1 and the HQ value exceeding 1 indicates a potential safety concern.

To better understand the impact of halocarbons on non-carcinogenic health risks in the YRD, the results from this study were compared with findings from other regions (Figure 4). The Shanxi site also displayed lower health risks in contrast with other regions. However, within the YRD (i.e., Hangzhou and Shanxi), 1,2-dichloropropane and trichloroethylene showed relatively higher non-carcinogenic risks and should be prioritized for future control. Also, the levels of chloroform and chloromethane at the Shanxi site showed a yearly increasing trend. These two halocarbons are widely used as solvents and chemical intermediates in industrial production [17,87].

The data from Beijing [13], Zhengzhou [88], Hangzhou [89], and Hong Kong, China [90] are cited in this work. The red line represents a value of 1.

Although the non-carcinogenic risks associated with halocarbons were comparatively minimal at the Shanxi site, local carcinogenic risks remained a significant concern. As shown in Figure S6, none of the species exceeded the tolerable risk level (1.0 × 10^−4^), though several substances surpassed the acceptable risk threshold (1.0 × 10^−6^). Among these, chloroform and 1,2-dichloroethane displayed carcinogenic risks persistently exceeding acceptable levels, with risk ranges of 1.20 × 10^−6^–4.03 × 10^−6^ and 6.41 × 10^−6^–1.13 × 10^−5^, respectively. 1,2-dibromoethane and carbon tetrachloride only exceeded acceptable levels in specific years. Despite declining concentration trends, the atmospheric lifetime of carbon tetrachloride is longer than the lifetimes of chloroform and 1,2-dichloroethane, which can prolong the exposure time compared to non-regulated halocarbons.

**Figure 5:**
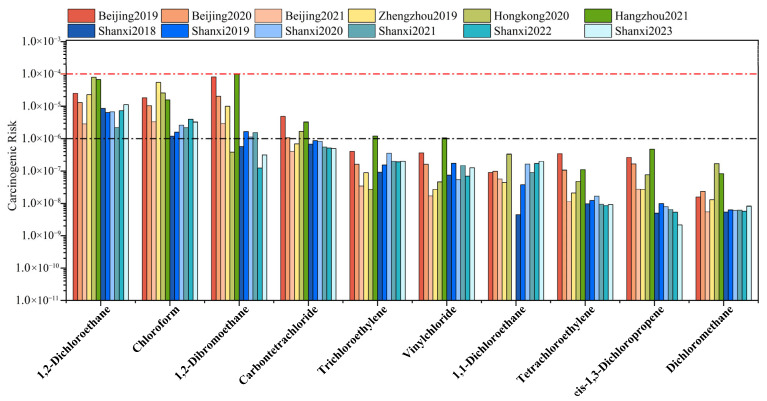
Comparison of the carcinogenic risk of selected halocarbons between Shanxi site and other studies. The data from Beijing [13], Zhengzhou [88], Hangzhou [89] and Hong Kong, China [90] are cited in this work. The red and black dotted lines at 10^−6^ and 10^−4^ represent the acceptable and tolerable levels of carcinogenic risk, respectively.

Figure 5 presents the comparative carcinogenic risk levels of selected halocarbons for this study and other areas. Although the YRD is one of major emission areas for halocarbons, carcinogenic risks at the Shanxi site remain lower than those in other Chinese cities, indicating regional heterogeneity in halocarbon emissions in the YRD. In other YRD regions, 1,2-dibromoethane demonstrated the highest carcinogenic risk (1.01 × 10^−4^), while carbon tetrachloride also exceeded acceptable levels. Consequently, the potential impacts from air mass transport originating in these areas require continuous consideration. At the Shanxi site, the carcinogenic risks of trichloroethylene (9.23 × 10^−8^–3.55 × 10^−7^) and vinyl chloride (5.44 × 10^−8^–1.73 × 10^−7^) surpassed those in Zhengzhou and Hong Kong. These compounds also exceeded acceptable risk levels in Hangzhou (trichloroethylene: 1.20 × 10^−6^ and vinyl chloride: 1.05 × 10^−6^). Given their prevalent use as cleaning agents in dry-cleaning and chemical feedstocks [68,91], these halocarbons warrant particular attention.

The monthly patterns of 1,2-Dichloroethane and chloroform are consistent with total halocarbon concentrations (Figure S7). Although risks declined from June onward, these species exhibited the widest error bar. The post-September period emerged as the highest-risk interval, with lifetime carcinogenic risks reaching 5.54 × 10^−6^–9.53 × 10^−6^ for 1,2-dichloroethane and 2.32 × 10^−6^–3.98 × 10^−6^ for chloroform, establishing this timeframe as a critical monitoring priority. 1,2-Dibromoethane and carbon tetrachloride exhibited seasonal exceedances of acceptable risk thresholds, with peak carcinogenic risks observed during the June–July period (1,2-dibromoethane in July: 8.78 × 10^−7^; carbon tetrachloride in June: 1.16 × 10^−6^). These elevated risks are related to seasonal temperature variation.

**Figure 6:**
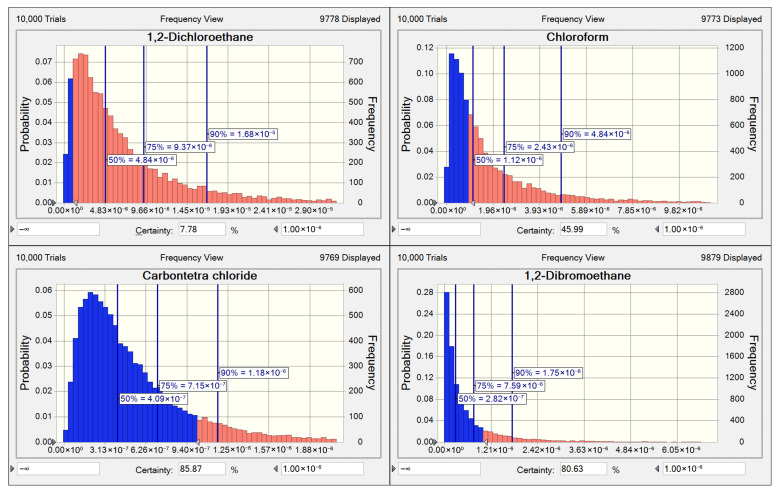
The distribution of carcinogenic risk of 4 halocarbons during the period of 2018–2023. The red shaded area represents the percentage of carcinogenic risk exceeding 1 × 10^−6^, while the blue area represents the percentage of risk below this threshold.

As shown in Figure 6, the 1,2-dichloroethane displayed the highest probability (92.2%) of exceeding the 1 × 10^−6^ carcinogenic risk threshold among these four compounds, with its 95th percentile risk value standing at 2.33 × 10^−5^. Chloroform demonstrated a 54.0% probability of surpassing acceptable levels across all years; however, its exceedance probability increased from 69.9% in 2021 to 80.0% in 2023 (Figure S8). It should be of concern at high concentrations. 1,2-dibromoethane and carbon tetrachloride showed lower exceedance probabilities (14.1% and 19.37%). Both compounds demonstrated declining trends in the probability of exceeding the 1 × 10^−6^ risk threshold during the study period (Figures S9 and S10). Nevertheless, given that carbon tetrachloride is under regulated, investigation into its potential sources remains warranted.

#### 3.3.2. The Health Risk of Sources

**Figure 7:**
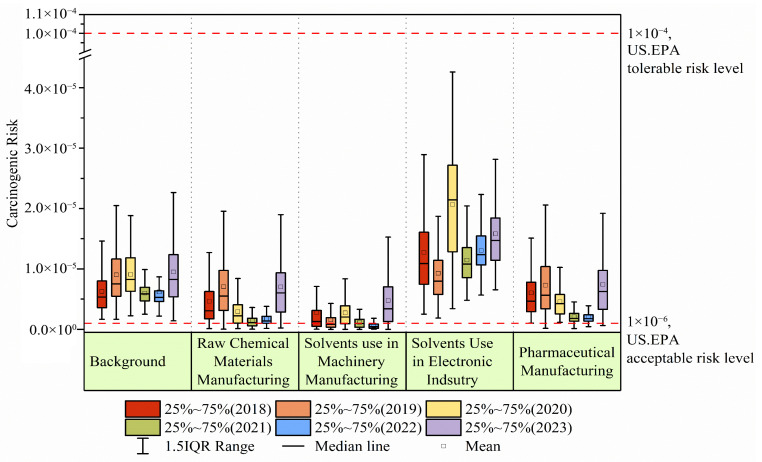
The carcinogenic risk of five halocarbon sources during the period of 2018–2023. The two dashed lines at 10^−6^ and 10^−4^ represent the acceptable and tolerable levels of carcinogenic risk.

Non-carcinogenic risks from all emission sources were under safe thresholds (Figure S11). As shown in Figure 7, carcinogenic risks across sources were prominent conversely. Solvent use in the electronic industry posed the highest carcinogenic risk to humans (9.30 × 10^−6^–2.07 × 10^−5^), primarily driven by substantial contributions from chloroform and 1,2-dichloroethane. Previous reports have shown that chloroform and 1,2-dichloroethane are widely used in solvent application and can cause tumors at high dosages in liver and kidneys [92,93]. Background sources ranked second in terms of carcinogenic risk (5.99 × 10^−6^–1.15 × 10^−5^). The observed increase in the carcinogenic risk of background sources during 2023 may be attributable to fugitive emissions near the site. Carcinogenic risks from pharmaceutical manufacturing (2.01 × 10^−6^–8.51 × 10^−6^) and the manufacturing of raw chemical materials and (1.41 × 10^−6^–7.05 × 10^−6^) were comparable. The peak value in pharmaceutical manufacturing in 2023 likely reflects the increased use of chloroform as a feedstock [25,94]. Chemical feedstocks demonstrated a progressive upward carcinogenic risk trend, with comparatively lower levels during 2020–2022 (range: 1.75 × 10^−6^–2.95 × 10^−6^), potentially linked to pandemic-induced slowdowns in rubber/plastic-related chemical manufacturing.

**Figure 8:**
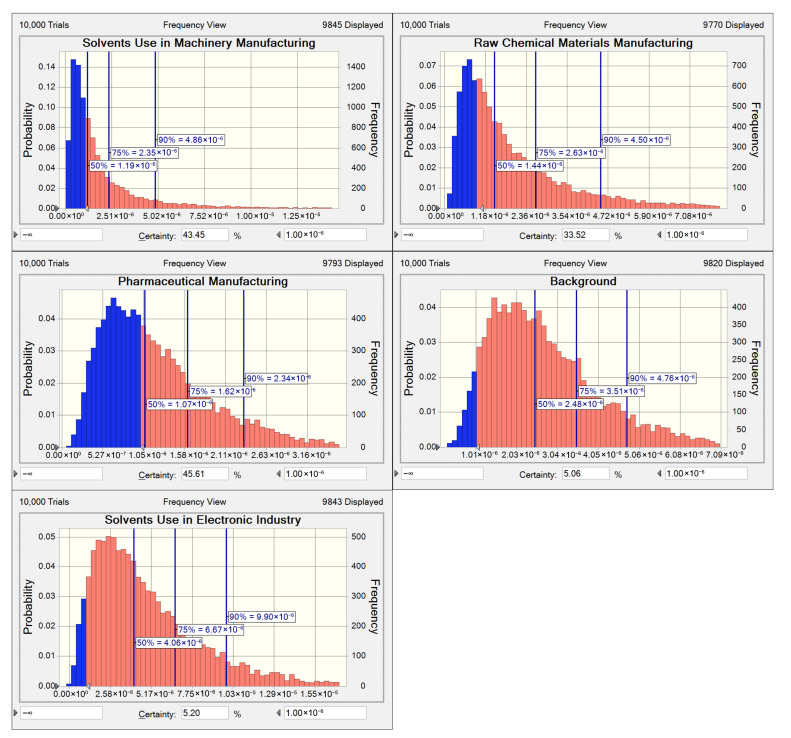
The distribution of the carcinogenic risk of five halocarbon sources during the period of 2018–2023. The red shaded area represents the percentage of carcinogenic risk exceeding 1 × 10^−6^, while the blue area represents the percentage of risk below this threshold.

Background sources and solvent use in electronic industry showed the highest probabilities of exceeding the acceptable risk threshold, reaching 94.9% and 94.8%, respectively (Figure 8). The 95th percentile carcinogenic risk value for solvent use in electronic industry reached 1.26 × 10^−5^, exceeding other sources by an order of magnitude. The probability of solvent use in the electronic industry exceeding the safety threshold remained within 85.2–97.3% across most years (Figure S12). Background sources demonstrated exceedance probabilities ranging from 12.9% to 90.5% (Figure S13), with increasing trends in both probability and 95th percentile risk values. Therefore, to minimize adverse health outcomes associated with halocarbons exposure, this study recommends implementing controls on emissions from solvent use in the electronic industry. Investigations should be conducted into the impacts of fugitive emission sources and the regional transport of halocarbons across the broader YRD region to inform subsequent risk management strategies.

## 4. Conclusions

This study conducted the continuous monitoring of 28 halocarbons at the Shanxi site in the central YRD from 2018 to 2023, systematically analyzing their temporal variations and source evolution patterns. The result show that halocarbon concentrations at the Shanxi site showed an overall increasing trend during the period of 2018–2023, with temporarily lower levels in seen 2020–2022 due to pandemic restrictions. Rising trends were primarily driven by elevated contributions from alkyl halides and alkenyl halides, notably dichloromethane, chloromethane, 1,2-dichloroethane, chloroform, and trichloroethylene. Furthermore, the PMF analysis revealed that pharmaceutical manufacturing is the dominant source with relatively stable contributions (37.0–60.2%). In contrast, the manufacturing of raw chemical materials (12.4–24.7%), solvent use in machinery manufacturing (8.0–19.9%) and electronic industry (10.3–13.7%) exhibit increasing contributions, predominantly traced to local emissions. Potential source areas of pharmaceutical manufacturing sources were related to southeastern local emissions, while background sources were primarily attributed to adjacent cities in the YRD region. Health risk assessment indicated that while overall health risks at the Shanxi site were relatively low, significant attention should be drawn to 1,2-dichloropropane, trichloroethylene, 1,2-dichloroethane, and chloroform due to their significant health risk. Additionally, solvent use in electronic industry and background sources are the dominant sources of health risk, with 94.9% and 94.8% probabilities of exceeding the acceptable risk threshold. These findings provide a scientific basis for formulating air quality management strategies, with relevance to the targeted abatement of halocarbon species and sources.

## Figures and Tables

**Table 1:** Comparison of halocarbons between Shanxi site and other studies in YRD region in 2018–2023 (Unit: ppb).

Site	Nanjing [30]	Hangzhou [29]	Hangzhou [28]	Shanxi (This Study)					
Duration	July 2018–August 2018	January 2021–December 2021	January 2021–February 2021	2018	2019	2020	2021	2022	2023
Dichloromethane	1.26	2.207	1.77	1.194 ± 0.891	1.440 ± 0.860	1.205 ± 1.139	1.351 ± 0.785	1.309 ± 0.817	1.831 ± 1.122
Chloromethane	0.16	0.912	0.66	0.401 ± 0.267	0.478 ± 0.423	0.205 ± 0.506	0.752 ± 0.694	0.666 ± 0.520	1.121 ± 0.721
Chloroform	0.17	0.129	0.11	0.082 ± 0.056	0.098 ± 0.120	0.294 ± 0.431	0.300 ± 0.314	0.299 ± 0.151	0.213 ± 0.088
Trichloroethylene	0.13	0.05	0.12	0.032 ± 0.027	0.064 ± 0.094	0.084 ± 0.132	0.122 ± 0.059	0.068 ± 0.051	0.070 ± 0.042
Tetrachloroethylene	0.06	0.057	0.07	0.043 ± 0.036	0.053 ± 0.036	0.064 ± 0.034	0.041 ± 0.023	0.037 ± 0.022	0.041 ± 0.030
1,2-Dichloroethane	0.95	0.596	0.71	0.632 ± 0.556	0.488 ± 0.532	0.399 ± 0.705	0.404 ± 0.297	0.632 ± 0.241	0.772 ± 0.354
1,2-Dichloropropane	0.57	0.306	0.24	0.217 ± 0.404	0.279 ± 0.358	0.267 ± 0.301	0.171 ± 0.151	0.099 ± 0.085	0.121 ± 0.113
Carbon tetrachloride	0.12	0.079	0.09	0.149 ± 0.079	0.186 ± 0.141	0.178 ± 0.101	0.118 ± 0.070	0.113 ± 0.076	0.109 ± 0.017
1,1-Dichloroethane	0.05	0.058	0.12	0.005 ± 0.004	0.034 ± 0.116	0.062 ± 0.265	0.417 ± 0.405	0.190 ± 0.302	0.217 ± 0.121
Vinyl chloride	0.05	0.043	0.05	0.027 ± 0.034	0.058 ± 0.063	0.020 ± 0.043	0.052 ± 0.080	0.025 ± 0.037	0.045 ± 0.057
1,2-Dibromoethane	0.18	0.02		0.001 ± 0.003	0.003 ± 0.008	0.002 ± 0.002	0.002 ± 0.002	0.000 ± 0.000	0.001 ± 0.001
cis-1,3-Dichloropropene	0.1	0.023	0.02	0.002 ± 0.006	0.003 ± 0.007	0.003 ± 0.004	0.003 ± 0.002	0.002 ± 0.012	0.001 ± 0.002
trans-1,3-Dichloropropene	0.13	0.017	0.02	0.001 ± 0.003	0.001 ± 0.002	0.001 ± 0.001	0.001 ± 0.001	0.002 ± 0.003	0.002 ± 0.002

## Data Availability

The raw data supporting the conclusions of this article will be made available by the corresponding authors on request.

## Supplementary Materials

The following supporting information can be downloaded at: https://www.mdpi.com/article/10.3390/toxics13121085/s1, Table S1. The Method Detection limit of TH-PKU 300B GC-MS/FID (Unit: ppb). Table S2. Meteorological parameters from 2018 to 2023. Table S3. Rfc and IUR values of halocarbon species in this study. Table S4. The input parameters in Monte Carlo simulation. Table S5. Distribution and parameters of adult outdoor exposure time in urban areas of Zhejiang province. Figure S1. The levels of halocarbons components at Shanxi site in 2018–2023. Figure S2. The month variation in halocarbons at Shanxi site in 2018–2023. Table S6. The characteristics and annual mean contributions of halocarbons at Shanxi site in 2018–2023 (Unit: ppb). Text S1. Source apportionment of halocarbons at Shanxi site. Figure S3. (a) Source profiles of PMF model analysis at Shanxi in 2018–2019. (b) Source profiles of PMF model analysis at Shanxi in 2020–2021. (c) Source profiles of PMF model analysis at Shanxi in 2022–2023. Text S2 Validation of 5-factor solutions for the PMF model. Table S7. The Qtrue/Qrobust and R2 of PMF model in 2018–2023. Text S3. The bootstrap results of six factors from the PMF model. Table S8. BS Test Results of Five Factors of PMF Model. Figure S4. Health index of halocarbons at Shanxi in 2018–2023. Figure S5. The yearly variation in non-carcinogenic risk of 19 halocarbons at Shanxi. Figure S6. The yearly variation in carcinogenic risk of 11 halocarbons at Shanxi. Figure S7 The monthly variation in the carcinogenic risk of 4 halocarbons at the Shanxi site. Figure S8. The yearly variation in carcinogenic risk of chloroform. Figure S9. The yearly variation in carcinogenic risk of carbon tetrachloride. Figure S10. The yearly variation in carcinogenic risk of 1,2-dibromoethane. Figure S11. The non-carcinogenic risk of five halocarbon sources during 2018–2023. Figure S12. The yearly variation in carcinogenic risk of solvent use in electronic industry. Figure S13. The yearly variation in carcinogenic risk of background (References [95,96,97,98,99,100,101,102,103,104,105,106,107,108,109,110,111,112,113,114,115,116,117,118] are cited in the supplementary materials).

## Abbreviations

The following abbreviations are used in this manuscript:

YRD	Yangtze River Delta
CFCs	Chlorofluorocarbons
HCFCs	Hydrochlorofluorocarbons
HFCs	Hydrofluorocarbons
ODSs	Ozone Depleting Substances
PMF	Positive Matrix Factorization
VOCs	Volatile Organic Compounds
CBPF	Conditional Bivariate Probability Function
EPA	U.S. Environmental Protection Agency
GC	Gas Chromatography
FID	Flame Ionization Detection
PLOT	Porous Layer Open Tubular
MS	Mass Spectrometric
