# Supplementary material for: Temporal Evolution, Source Apportionment, and Health Risks of Atmospheric Halocarbons: A Case Study in the Central Yangtze River Delta Region
Source: Toxics. 2025 Dec 17;13(12):1085. doi: 10.3390/toxics13121085 (PMC12737745; doi:10.3390/toxics13121085)
Supplement: Supplementary file 1 [file toxics-13-01085-s001.zip › toxics-3978531-supplementary.pdf]

Supplementary Information

Temporal Evolution, Source Apportionment, and Health Risks of Atmospheric Halocarbons: A Case Study in the Central Yangtze River Delta Region

Table S1 The Method Detection limit of TH-PKU 300B GC-MS/FID (Unit: ppb)

Species	MDLs	Species	MDLs
Chloromethane	0.063	Bromodichloromethane	0.037
Dichloromethane	0.011	1,2-Dibromoethane	0.009
Chloroform	0.021	Vinyl chloride	0.039
1,2-Dichloropropane	0.007	Trichloroethylene	0.013
1,2-Dichloroethane	0.043	Tetrachloroethylene	0.017
Chloroethane	0.019	cis-1,3-Dichloropropene	0.019
CFC-11	0.008	trans-1,3-Dichloropropene	0.02
CFC-113	0.008	cis-1,2-Dichloroethene	0.031
CFC-114	0.103	1,1-Dichloroethene	0.024
Carbon tetrachloride	0.013	Benzyl chloride	0.044
1,1-Dichloroethane	0.02	chlorobenzene	0.009
1,1,2-trichloroethane	0.008	1,3-Dichlorobenzene	0.016
Bromomethane	0.02	1,4-Dichlorobenzene	0.019
1,1,1-Trichloroethane	0.053	1,2-Dichlorobenzene	0.009

Table S2 Meteorological parameters from 2018 to 2023.

Sampling period	Temperature (°C)	Relative humidity (%)	Pressure (hPa)	Wind speed (m/s)
2018	17.42	80.04	1015.79	2.40
2019	17.63	79.78	1015.38	2.29
2020	18.13	79.99	1015.69	2.24
2021	18.41	78.39	1015.38	2.29
2022	17.80	79.13	1015.55	2.33
2023	17.94	79.26	1016.07	2.27

Lufft WS500-UMB can measure wind direction and speed insusceptibly to bird nesting. Their compact weather sensor combines 4 measurement parameters in one housing with only one cable connection, minimizing installation complexity and maintenance needs.

Table S3 Rfc and IUR values of halocarbon species in this study

Species	Rfc (mg/m^3)	IUR ($m^3/\mu g$)	Species	Rfc (mg/m^3)	IUR ($m^3/\mu g$)
Chloromethane	9.00×10^{-2}		chlorobenzene	1.00×10^{0}	
Bromomethane	5.00×10^{-3}		1,2-Dichloroethane	2.40×10^{0}	2.60×10^{-5}
Chloroethane	1.00×10^{1}		1,2-Dichloropropane	4.00×10^{-3}	
Vinyl chloride	1.00×10^{-1}	8.8×10^{-6}	cis-1,3-Dichloropropene	2.00×10^{-2}	4.00×10^{-6}
Trichloroethylene	2.00×10^{-3}	4.10×10^{-6}	1,1,1-Trichloroethane	5.00×10^{-0}	
1,1-Dichloroethene	2.00×10^{-1}		1,1,2-trichloroethane	4.00×10^{-1}	1.60×10^{-5}
Dichloromethane	6.00×10^{-1}	1.00×10^{-8}	Tetrachloroethylene	4.00×10^{-2}	2.60×10^{-7}
Chloroform	9.80×10^{-2}	2.30×10^{-5}	1,4-Dichlorobenzene	8.00×10^{-1}	
Carbon tetrachloride	1.00×10^{-1}	6.00×10^{-6}	1,1-Dichloroethane	5.00×10^{-1}	1.60×10^{-6}
1,2-Dibromoethane	9.00×10^{-3}	6.00×10^{-4}			

Table S4 The input parameters in Monte Carlo simulation

Parameter	Units	Distribution	Distribution parameters	References
Exposure concentration	μg/m³	Lognormal	Fit distribution to data	Observation
Exposure frequency	d/a	Triangular	min=180, max=345, μ=345	[1]
Exposure time	min/d	Lognormal	Table S5	[2]
Exposure duration	a	Constant	77.73	[2]
Average time	h	Constant	680914.8	[2]
IUR	m³/μg	Constant	Table S3	IRIS and OEHHA
Rfc	mg/m³	Constant	Table S3	IRIS

Table S5 Distribution and parameters of adult outdoor exposure time in urban areas of Zhejiang province

Parameters	Distribution	Mean	P5	P25	P50	P75	P95
Exposure time (min/d)	Lognormal	199	45	100	161	257	477

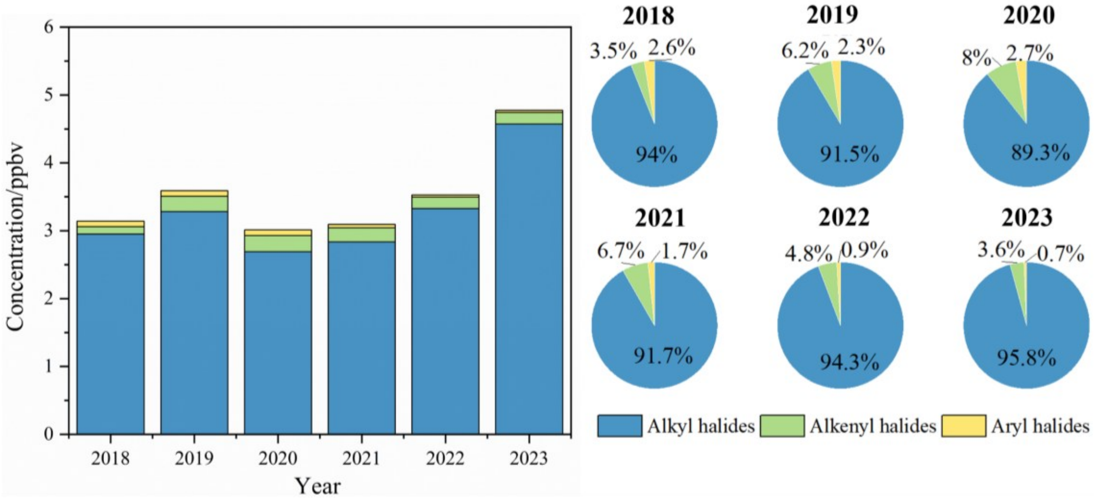

Fig. S1 The levels of halocarbons components at Shanxi site in 2018-2023

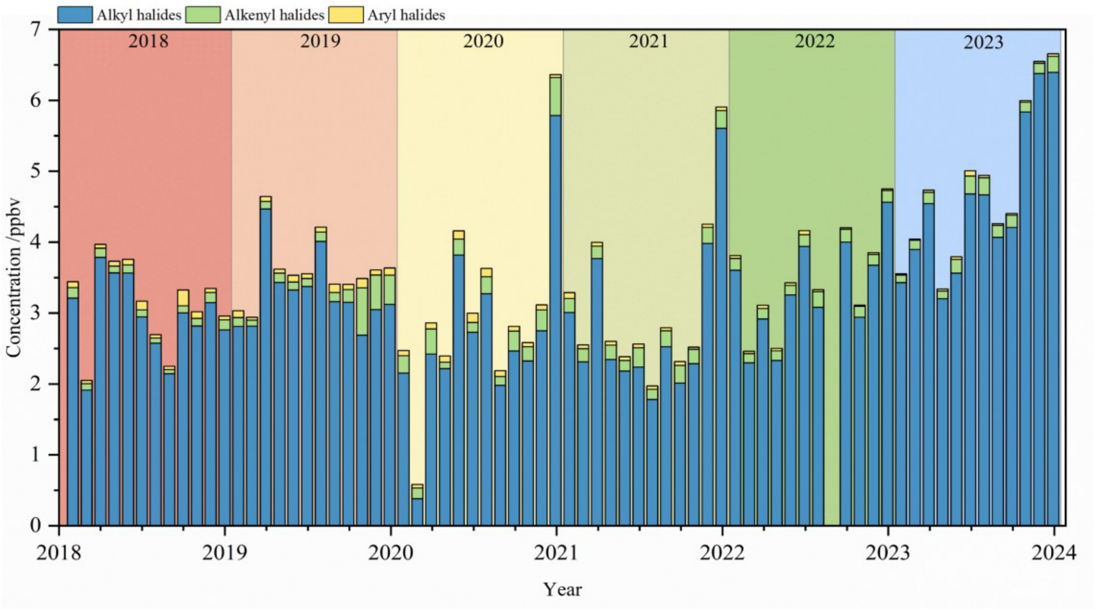

Fig. S2 The month variation of halocarbons at Shanxi site in 2018-2023

Table S6 The characteristics and annual mean contributions of halocarbons at Shanxi site in 2018-2023 (Unit: ppb)

Species	2018	2019	2020	2021	2022	2023	%
Freon114	0.010±0.006	0.009±0.006	0.013±0.005	0.017±0.006	0.013±0.006	0.015±0.003	0.30%
Chloromethane	0.401±0.267	0.478±0.423	0.205±0.506	0.752±0.694	0.666±0.520	1.121±0.721	14.40%
Bromomethane	0.026±0.021	0.027±0.020	0.018±0.010	0.027±0.020	0.022±0.024	0.025±0.020	0.60%
Chloroethane	0.026±0.031	0.036±0.035	0.029±0.021	0.027±0.028	0.039±0.041	0.055±0.060	0.90%
Freon11	0.355±0.138	0.394±0.150	0.381±0.194	0.232±0.098	0.338±0.143	0.269±0.013	8.50%
Freon113	0.058±0.010	0.065±0.012	0.063±0.014	0.072±0.014	0.069±0.011	0.073±0.008	1.70%
Dichloromethane	1.194±0.891	1.440±0.860	1.205±1.139	1.351±0.785	1.309±0.817	1.831±1.122	34.70%
1,1-Dichloroethane	0.005±0.004	0.034±0.116	0.062±0.265	0.417±0.405	0.190±0.302	0.217±0.121	3.60%
Chloroform	0.082±0.056	0.098±0.120	0.294±0.431	0.300±0.314	0.299±0.151	0.213±0.088	5.40%
1,1,1-Trichloroethane	0.002±0.001	0.003±0.005	0.003±0.001	0.003±0.002	0.002±0.003	0.001±0.001	0.10%
Carbon tetrachloride	0.149±0.079	0.186±0.141	0.178±0.101	0.118±0.070	0.113±0.076	0.109±0.017	3.70%
1,2-Dichloroethane	0.632±0.556	0.488±0.532	0.399±0.705	0.404±0.297	0.632±0.241	0.772±0.354	13.90%
1,2-Dichloropropane	0.217±0.404	0.279±0.358	0.267±0.301	0.171±0.151	0.099±0.085	0.121±0.113	5.10%
Bromodichloromethane	0.001±0.001	0.002±0.002	0.000±0.000	0.000±0.000	0.001±0.001	0.002±0.001	0.70%
1,1,2-trichloroethane	0.015±0.029	0.031±0.072	0.018±0.052	0.048±0.042	0.030±0.022	0.026±0.031	0.00%
1,2-Dibromoethane	0.001±0.003	0.003±0.008	0.002±0.002	0.002±0.002	0.000±0.000	0.001±0.001	0.00%
Vinyl chloride	0.027±0.034	0.058±0.063	0.020±0.043	0.052±0.080	0.025±0.037	0.045±0.057	0.90%
1,1-Dichloroethene	0.003±0.003	0.021±0.039	0.024±0.016	0.018±0.023	0.008±0.024	0.007±0.007	0.30%
cis-1,2-Dichloroethene	0.001±0.002	0.023±0.059	0.035±0.030	0.023±0.026	0.023±0.089	0.008±0.007	0.50%

Trichloroethylene	0.032±0.027	0.064±0.094	0.084±0.132	0.122±0.059	0.068±0.051	0.070±0.042	1.80%
cis-1,3-Dichloropropene	0.002±0.006	0.003±0.007	0.003±0.004	0.003±0.002	0.002±0.012	0.001±0.002	0.10%
trans-1,3-Dichloropropene	0.001±0.003	0.001±0.002	0.001±0.001	0.001±0.001	0.002±0.003	0.002±0.002	0.00%
Tetrachloroethylene	0.043±0.036	0.053±0.036	0.064±0.034	0.041±0.023	0.037±0.022	0.041±0.030	1.20%
chlorobenzene	0.034±0.048	0.050±0.070	0.054±0.071	0.024±0.026	0.018±0.017	0.017±0.014	0.90%
1,3-Dichlorobenzene	0.006±0.021	0.005±0.007	0.005±0.003	0.005±0.005	0.002±0.003	0.002±0.007	0.10%
1,2-Dichlorobenzene	0.009±0.016	0.009±0.008	0.010±0.007	0.009±0.006	0.005±0.005	0.006±0.010	0.20%
1,4-Dichlorobenzene	0.015±0.026	0.016±0.014	0.012±0.006	0.010±0.004	0.007±0.005	0.007±0.009	0.30%
Benzyl chloride	0.003±0.007	0.002±0.003	0.004±0.004	0.003±0.003	0.001±0.002	0.001±0.004	0.10%

Text S1. Source apportionment of halocarbons at Shanxi site

Factor 1 is characterized by high percentage contributions from 1,2-dichloropropane and 1,2-dichloroethane, with their contributions to this source varying in the ranges of 63.0% - 88.0% and 5.1% - 33.1%, respectively. These two compounds are widely used as solvent components in coatings for mechanical manufacturing [3]. Additionally, 1,2-dichloropropane and 1,2-dichloroethane are also utilized as diluents or spray solvents in the automotive repair industry [4,5]. Furthermore, during the period of 2020 - 2023, dichloromethane and tetrachloroethylene were allocated to this source at relatively high proportions, ranging from 28.4% to 46.7% and 25.4% to 37.8%, respectively. Dichloromethane is also an effective paint stripper in the mechanical manufacturing industry, while tetrachloroethylene is a commonly used degreaser for metal surfaces in industrial applications [6,7]. Based on the above information, Factor 1 is identified as solvent use in mechanical manufacturing.

Factor 2 is characterized by a high percentage contribution from methyl chloride, with its contribution ranging from 29.8% to 100.0% over the years. During 2018 - 2019, methyl chloride, along with dichloromethane (41.3% - 41.3%) and trichloromethane (20.4% - 35.5%), all contributed significantly to this source. This is attributed to the chlor-alkali industry, where methyl chloride plants produce or emit large amounts of these methyl chlorides [8,9]. Therefore, during 2018 - 2019, the dominant industry in factor 2 is chemical raw material manufacturing in which the raw material is methyl chloride. In China, the emissions from raw material manufacturing of methyl chloride decreased continuously during 2018 - 2019, while the emissions of methyl chloride used as a solvent in other chemical raw material manufacturing industries showed an increasing trend [10]. In this study, the concentration of methyl chloride increased year by year, consistent with the trend of increasing solvent usage in other chemical raw material manufacturing processes. Given that methyl chloride and 1,3-butadiene have been identified as typical emission factors in the manufacturing of synthetic resins, rubber, or plastics [11,12], this study introduced 1,3-butadiene into the PMF model during 2020 - 2023, in order to indicate the possible sources of factor 2. The contribution percentage of 1,3-butadiene reached 16.9% - 51.2%. In addition, during 2020, 2021, and 2022, the proportions of vinyl chloride allocated to this source were 41.5%, 16.4%, and 65.4%, respectively. This substance is a precursor in the production process of polyvinyl chloride (PVC) resins [13].

Factor 3 is characterized by high percentage contributions from dichloromethane and acetone, with their respective contributions ranging from 5.4% to 54.5% and 34.1% to 88.3% during 2018 - 2023. In numerous studies focusing on the identification of industry-specific VOCs in pharmaceutical production, acetone and dichloromethane have been regarded as signature compounds[14,15]. According to the industrial emission inventory of China in 2018 compiled by Liang et al., acetone can account for up to 68.37% of emissions from medical manufacturing [11]. Lin et al. identified dichloromethane as one of the characteristic pollutants in pharmaceutical emissions during their investigation of representative VOCs from pharmaceutical plants [16]. Additionally, Zhong et al. reported that dichloromethane concentrations exceeded 12.5% in biopharmaceutical processes [17]. In this study, chlorobenzene and 1,4-dichlorobenzene also contributed significantly to this source during 2020 - 2023, with contribution percentages ranging from 25.4% to 66.1% and 9.4% to 67.9%, respectively. These compounds are the high-contributing halocarbons in the production of penicillin salts, vitamins, and amoxicillin [18]. Moreover, the "Emission Standard of Air Pollutants for the Pharmaceutical Industry" (DB33 310005-2021) lists chlorobenzene compounds as typical air pollutants emitted during the manufacturing processes of sulfonamide and potentiating agents [19]. Therefore, this source is associated with pharmaceutical manufacturing.

Factor 4 is characterized by a high proportion of regulated halocarbons, with CFC-11 and carbon tetrachloride being the most prominent, followed by CFC-113 and 1,1,1-trichloroethane. These four substances have average contribution proportions ranging from 37.8% to 66.1% over the six-year period. All of these compounds are regulated under the Montreal Protocol and have been banned for over a decade [20]. Before being regulated by protocol, CFC-11 was widely used as a refrigerant and propellant [21], CFC-113 was employed in cleaning agents [22], and 1,1,1-trichloroethane served as a degreasing agent in electronics manufacturing and as a chemical intermediate in the production of vinylidene chloride [23]. Additionally, 1,2-dibromoethane (ethylene dibromide) shows significant contributions in some years, such as 85.1% in 2019 and 65.0% in 2023. 1,2-Dibromoethane was historically used as an anti-knock additive in gasoline and as a soil fumigant [24], However, due to its high toxicity, the use of 1,2-dibromoethane as a pesticide has been banned in China [25]. Based on these characteristics, factor 4 is identified as a background source.

Factor 5 is characterized by high proportions of trichloroethylene, chloroform, and 1,1-dichloroethane, with their respective contributions to this source ranging from 53.40% to 86.18%, 40.38% to 76.52%, and 34.38% to 94.87% over the entire study period. Numerous studies have shown that trichloroethylene is a commonly used degreasing agent in the electronics and precision instrument cleaning industries [26,27]. Monitoring of the electronics industry in other YRD region, revealed that trichloroethylene is one of the major pollutants in the printed circuit board manufacturing industry [28] and during the pre-treatment of substrates and photolithography processes in optoelectronic display manufacturing [29]. Chloroform is an important component of solvent-based cleaning agents used in the electronics manufacturing industry [30]. 1,1-Dichloroethane, a good solvent for plastics, oils, and fats, is often used as a cleaning agent for electronic components and metals, as a degreasing agent [31]. Based on these findings, this source is related to cleaning agents used in the electronics manufacturing industry.

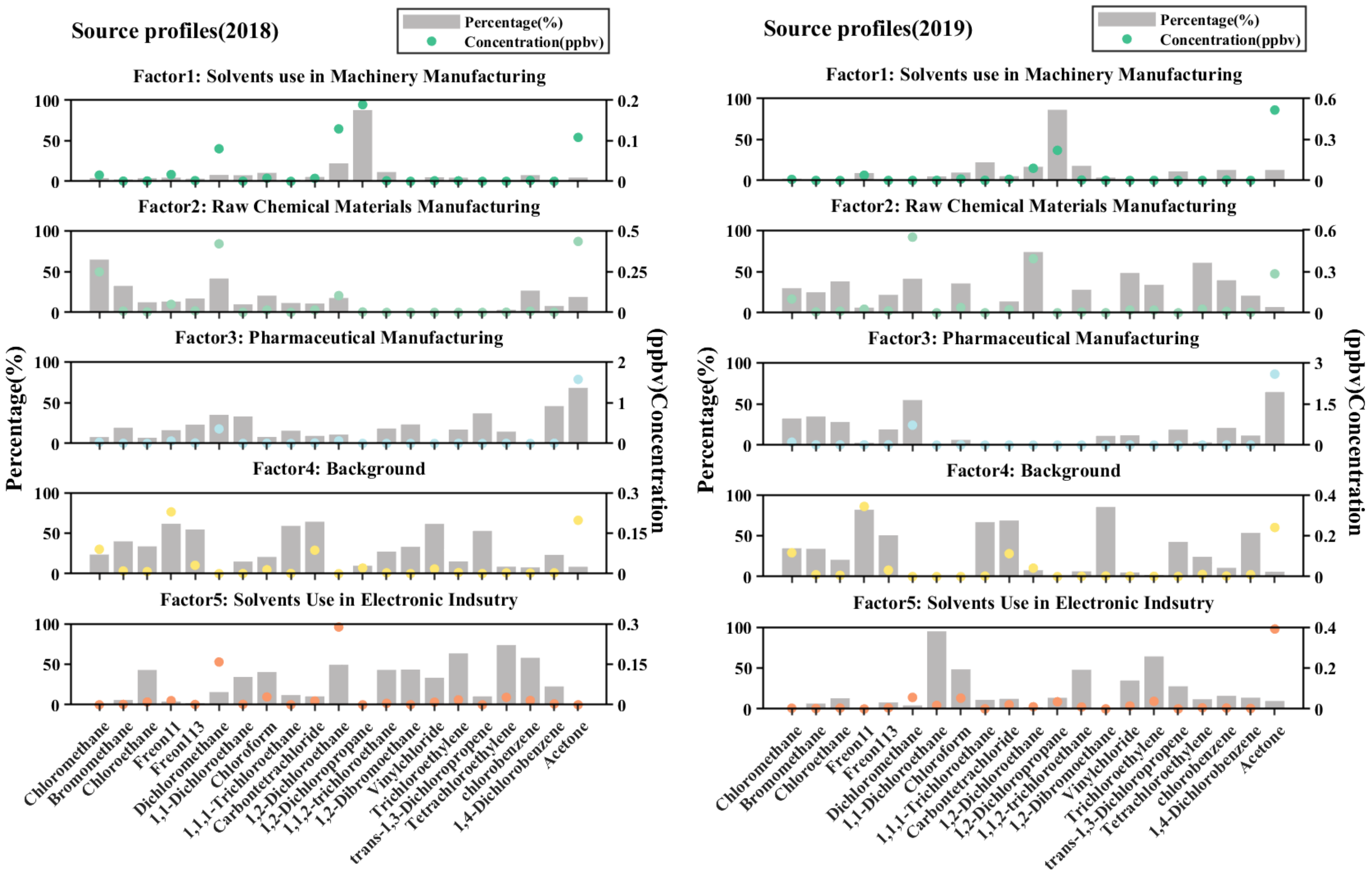

Fig. S3 (a)Source Profiles of PMF Model Analysis at Shanxi in 2018-2019

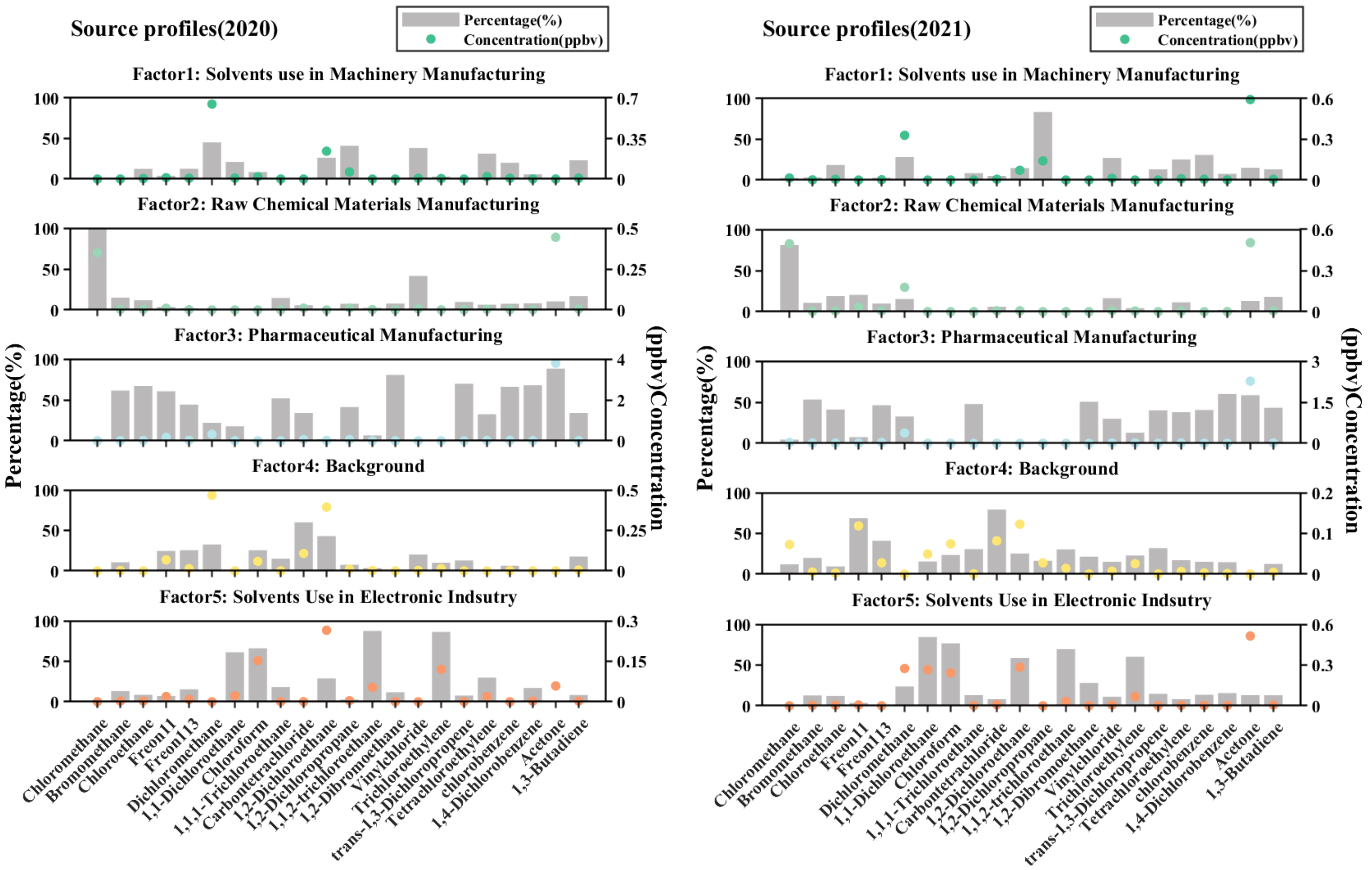

Fig. S3 (b) Source Profiles of PMF Model Analysis at Shanxi in 2020-2021

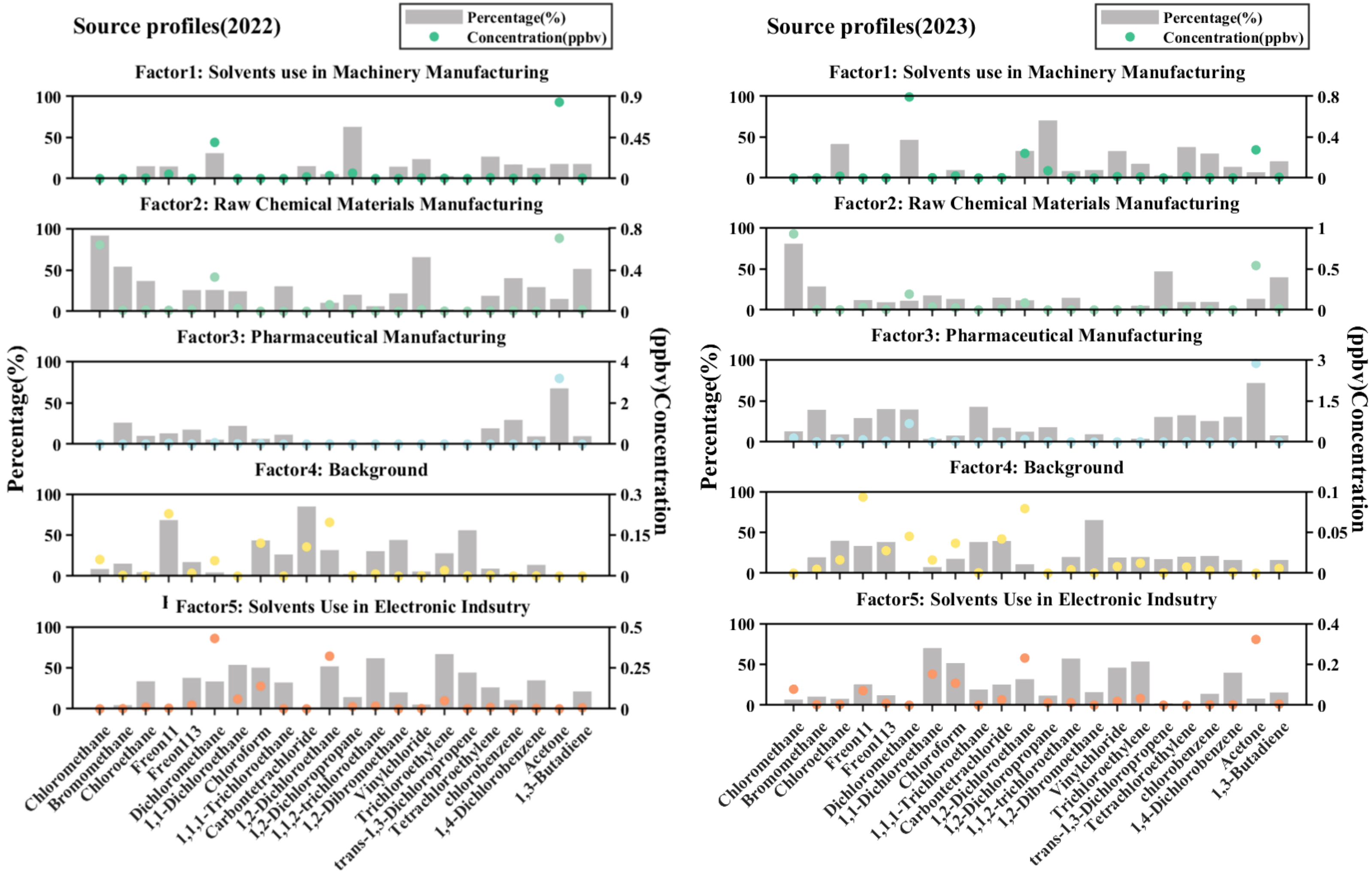

Fig. S3 (c) Source Profiles of PMF Model Analysis at Shanxi in 2022-2023

Text S2 Validation of 5-factor solutions for the PMF modle

Based on the species selection criteria mentioned in previous studies [32], 20 halocarbon species, acetone, and 1,3-butadiene were selected as input species. Among all the PMF model runs, a five-factor solution was chosen as the optimal number of contributing factors. In this study, the ratio of Q_{true} to Q_{robust} in the final solutions for all years consistently met the condition of being less than 1.5 and close to 1, which confirms the rationality of the selected number of source factors (EPA/600/R-14/108). Additionally, a correlation analysis was conducted between the modeled total concentrations and the observed concentrations. The results showed that the R^2 values for all six years were greater than 75%, indicating that the source apportionment results obtained in this study are reliable. Table S7 presents the detailed Q_{true}/Q_{robust} ratios and R^2 values for the final PMF model runs in each year.

Table S7 The Q_{true}/Q_{robust} and $R^2$of PMF model in 2018-2023

Year	Q_{true}/Q_{robust}	R^2
2018	1.00	91.93%
2019	0.99	92.17%
2020	0.97	75.58%
2021	0.98	82.04%
2022	0.99	95.61%
2023	0.98	93.10%

Text S3 The bootstrap results of six factors from the PMF model

This study also conducted a validation of the model feasibility for the results obtained from the PMF model, following the Bootstrap method as described in the EPA PMF 5.0 User's Guide (EPA/600/R-14/108). This method involves resampling the original dataset with replacement to create datasets of the same dimension as the original dataset, which are then input into the model to assess the impact of random errors and specific observations on the PMF solution. A total of 100 Bootstrap runs were performed in this study, with a minimum correlation setting of 0.6.

Table S8 BS Test Results of Five Factors of PMF Model

Year	Factor name	Solvents use in Machinery Manufacturing	Raw Chemical Materials Manufacturing	Pharmaceutical Manufacturing	Background	Solvents Use in Electronic Industry	Unmapped
	Boot Factor 1	100	0	0	0	0	0
	Boot Factor 2	0	100	0	0	0	0
2018	Boot Factor 3	0	0	100	0	0	0
	Boot Factor 4	0	0	0	100	0	0
	Boot Factor 5	0	0	4	0	100	0
	Boot Factor 1	0	0	100	0	0	0
	Boot Factor 2	0	0	0	100	0	0
2019	Boot Factor 3	1	99	0	0	0	0
	Boot Factor 4	100	0	0	0	0	0
	Boot Factor 5	0	4	0	0	96	0
	Boot Factor 1	0	100	0	0	0	0
	Boot Factor 2	0	0	0	99	1	0
2020	Boot Factor 3	0	0	0	0	100	0
	Boot Factor 4	20	0	80	0	0	0
	Boot Factor 5	23	9	0	66	2	0
	Boot Factor 1	100	0	0	0	0	0
	Boot Factor 2	0	0	100	0	0	0
2021	Boot Factor 3	0	100	0	0	0	0
	Boot Factor 4	0	0	0	0	100	0
	Boot Factor 5	0	0	0	100	0	0
	Boot Factor 1	100	0	0	0	0	0
	Boot Factor 2	0	0	0	100	0	0
2022	Boot Factor 3	0	100	0	0	0	0
	Boot Factor 4	0	3	0	0	88	0
	Boot Factor 5	0	0	100	0	0	0
	Boot Factor 1	8	0	0	90	0	2
	Boot Factor 2	100	0	0	0	0	0
2023	Boot Factor 3	4	0	96	0	0	0
	Boot Factor 4	0	100	0	0	0	0
	Boot Factor 5	0	0	0	1	99	0

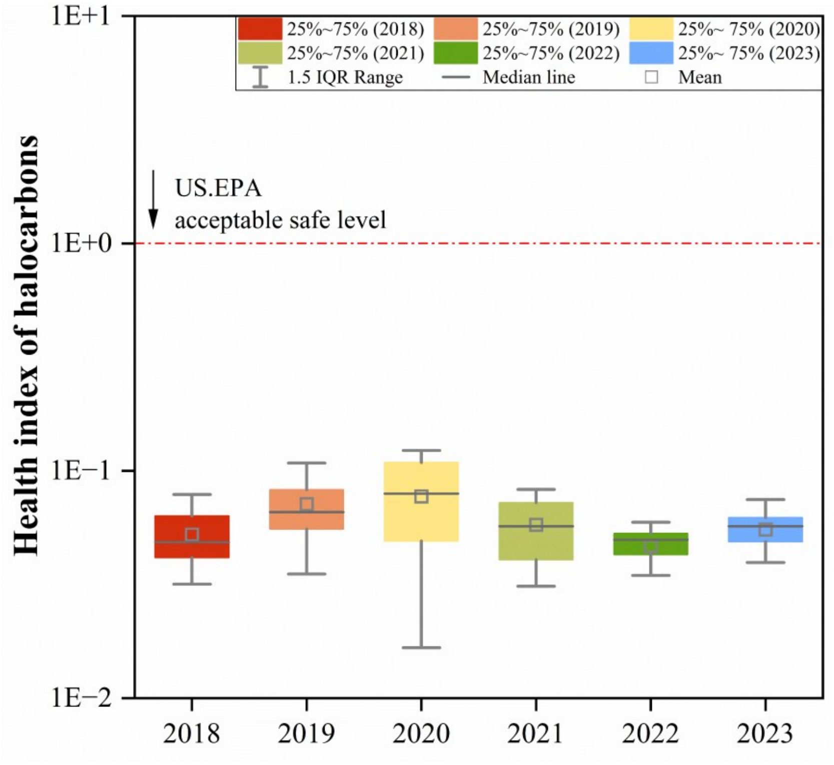

Fig. S4 Health index of halocarbons at Shanxi in 2018-2023

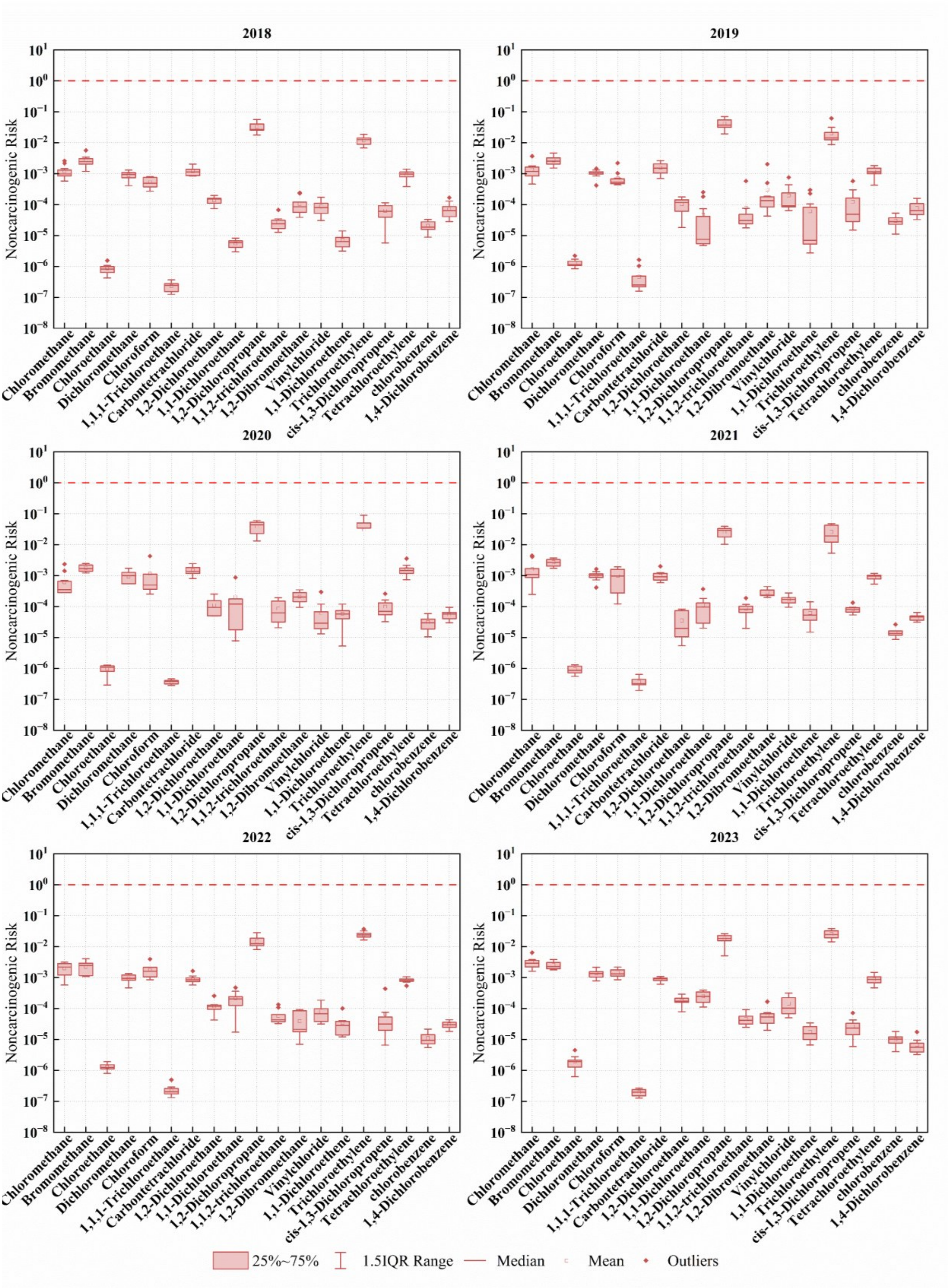

Fig. S5 The yearly variation of non-carcinogenic risk of 19 halocarbons at Shanxi

The red line represents 1.

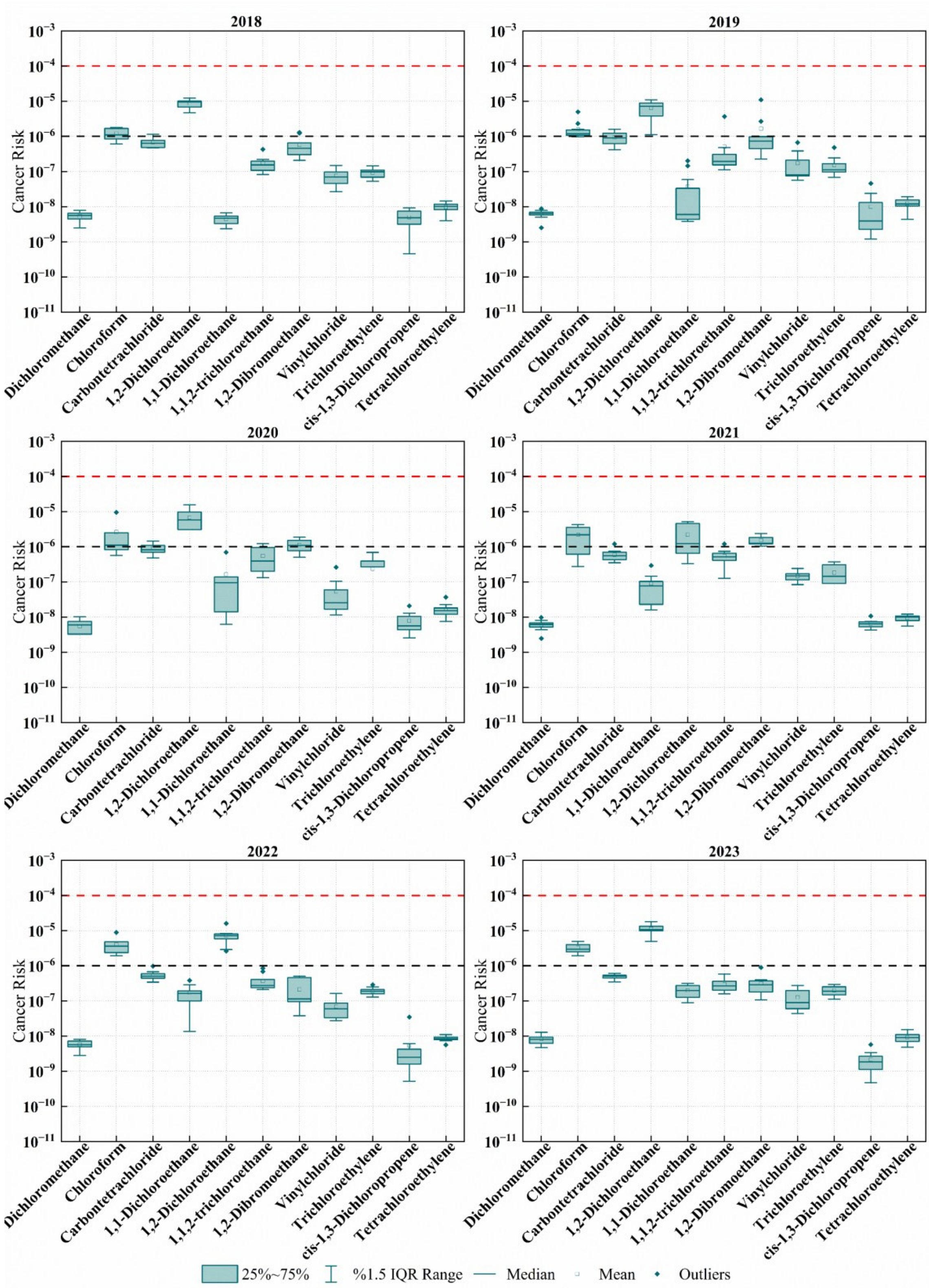

Fig. S6 The yearly variation of carcinogenic risk of 11 halocarbons at Shanxi

The two dashed lines at 10^{-6} and 10^{-4} represent the acceptable and tolerable levels of carcinogenic risk.

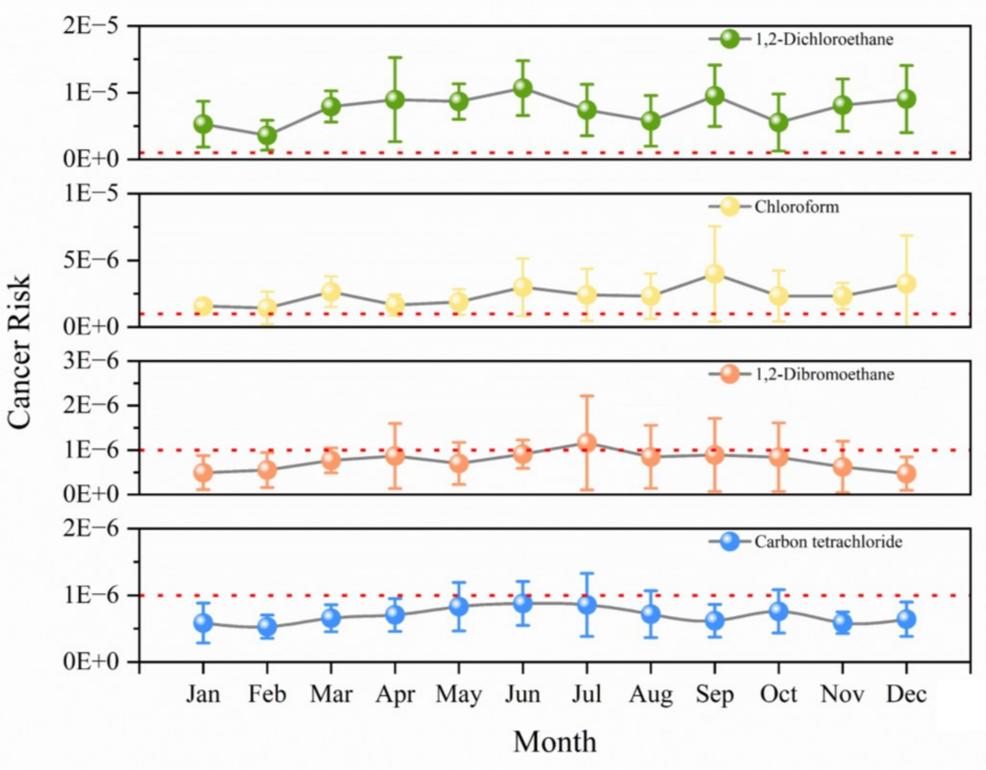

Fig. S7 The Monthly Variation of Carcinogenic Risk of 4 Halocarbons at Shanxi site

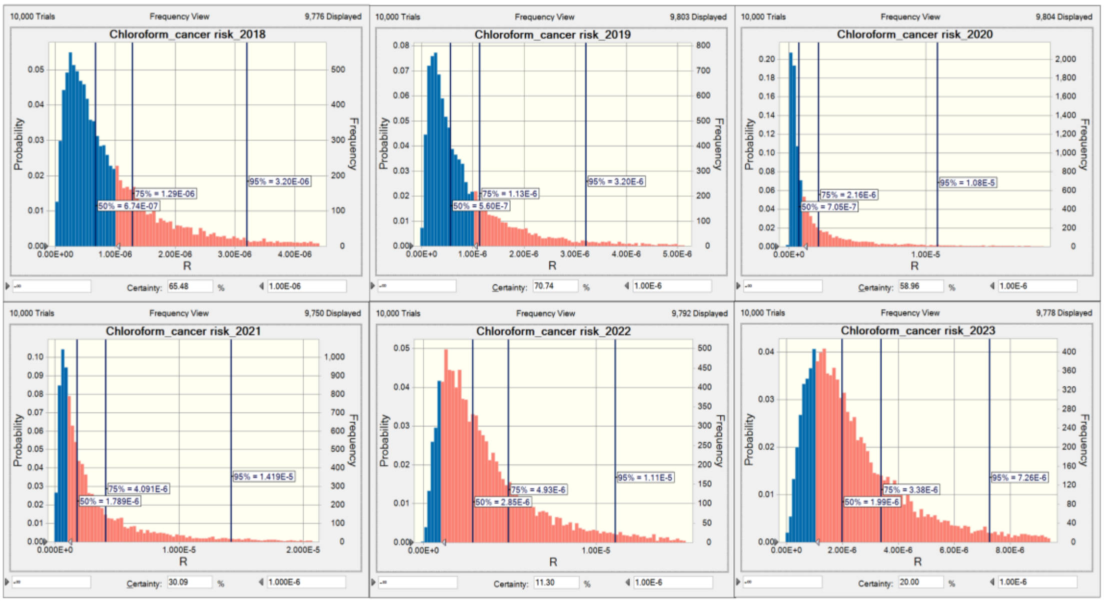

Fig. S8 The yearly variation of carcinogenic risk of chloroform

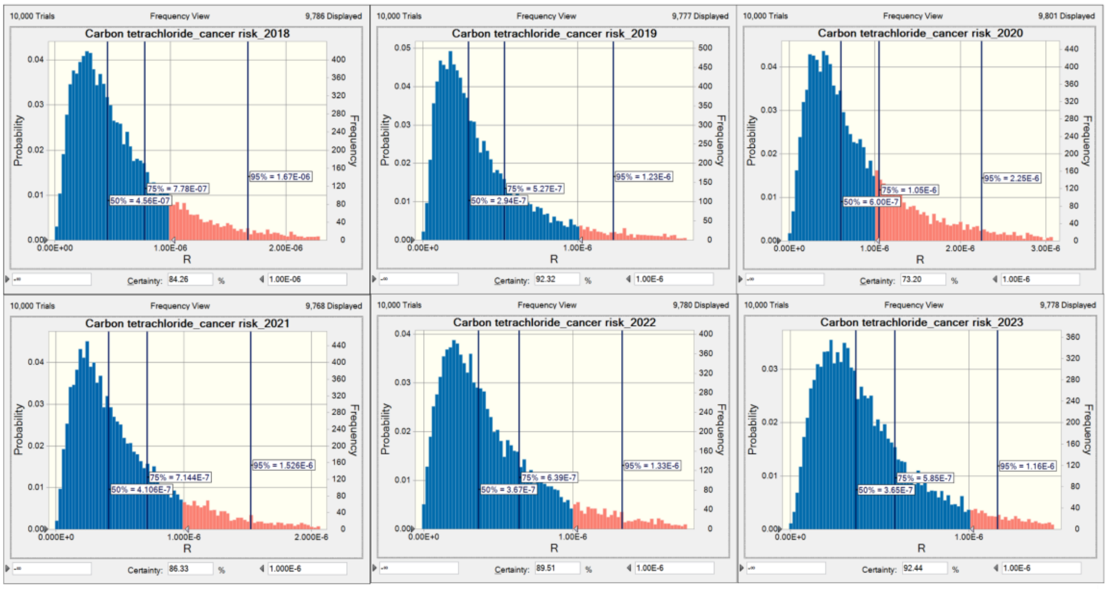

Fig. S9 The yearly variation of carcinogenic risk of carbon tetrachloride

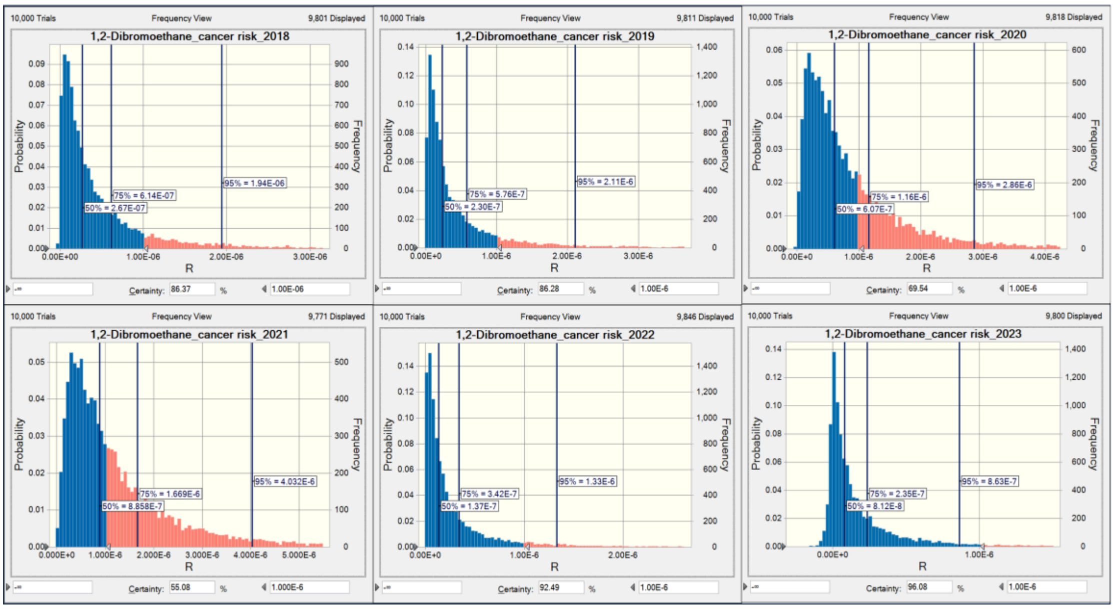

Fig. S10 The yearly variation of carcinogenic risk of 1,2-dibromoethane

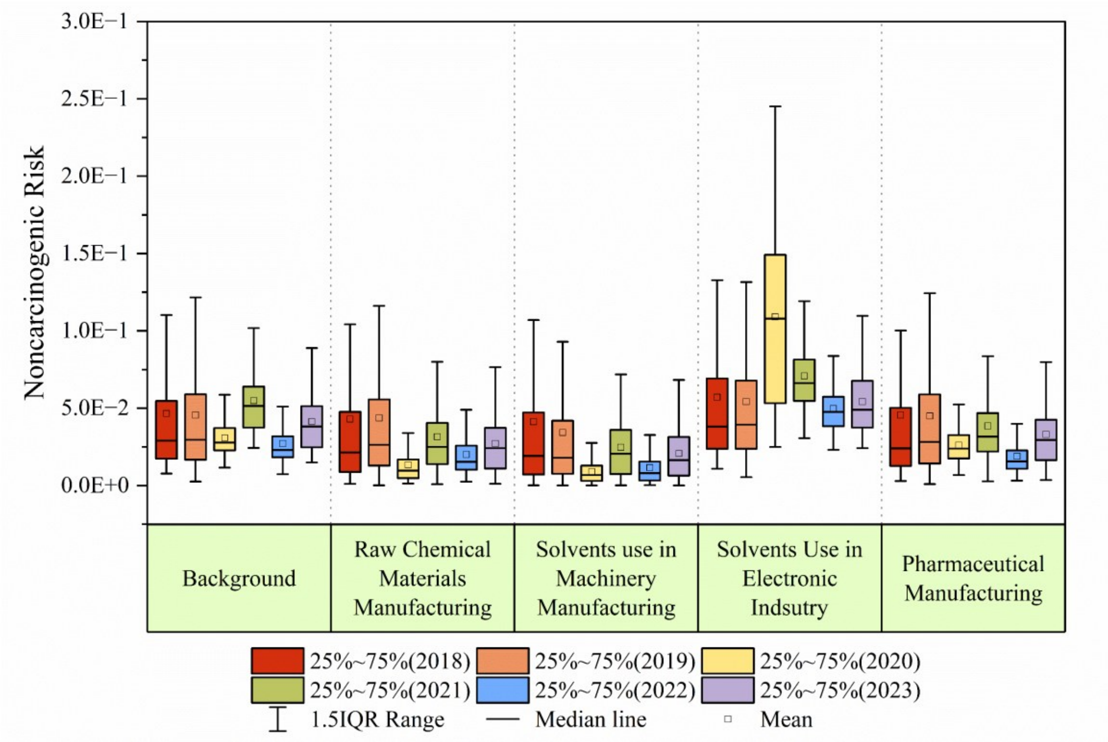

Fig. S11 The non-carcinogenic risk of five halocarbon sources during 2018-2023

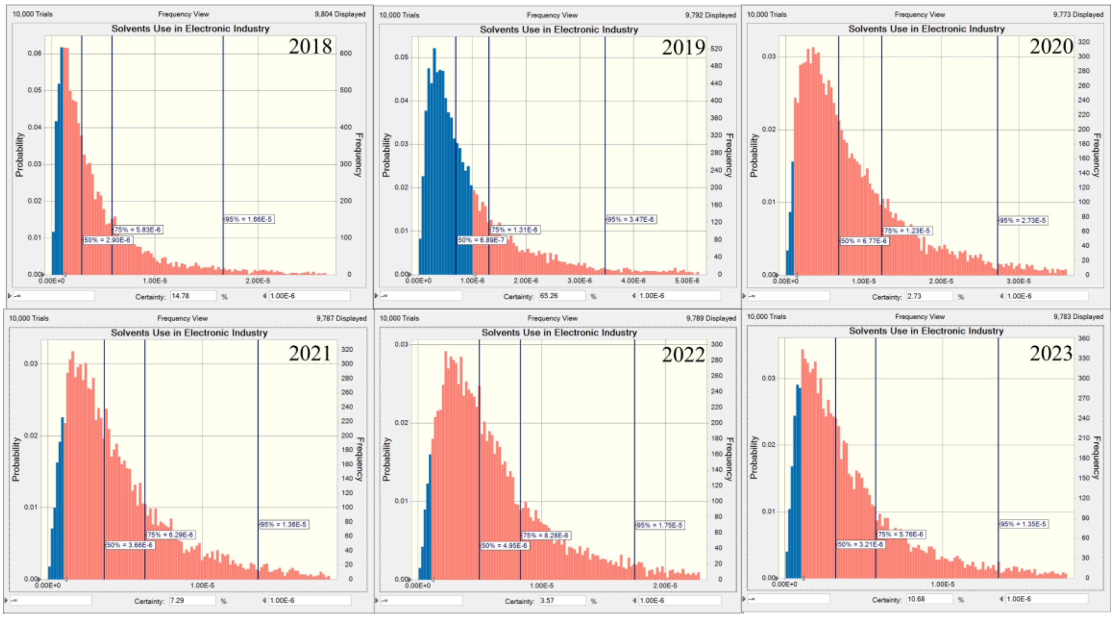

Fig. S12 The yearly variation of carcinogenic risk of solvents use in electronic industry

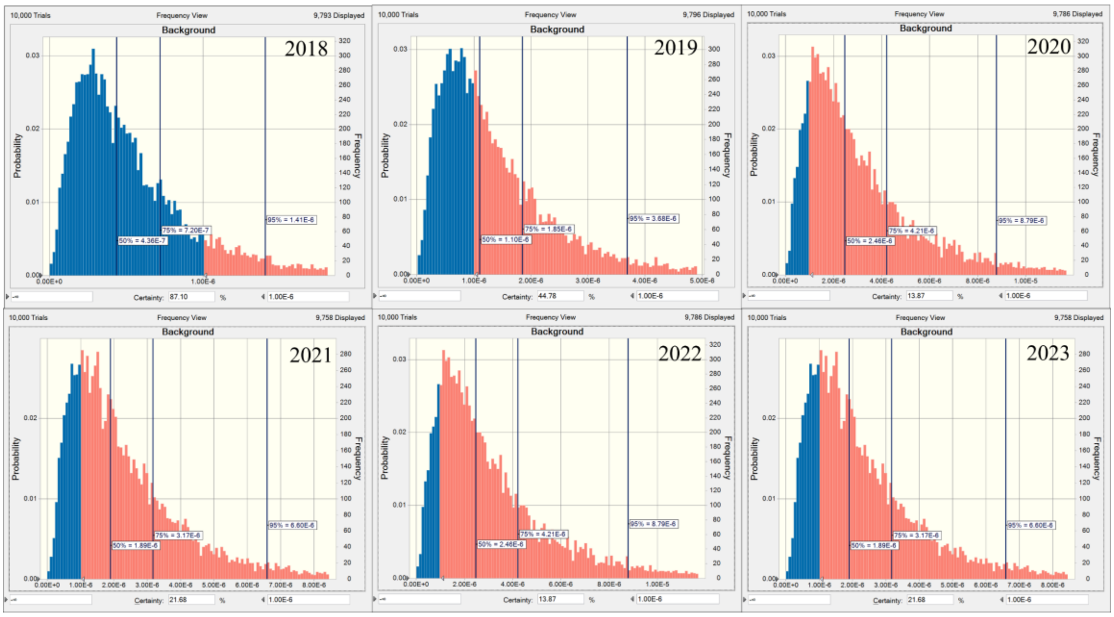

Fig. S13 The yearly variation of carcinogenic risk of background

References

1. Smith, R.L. Use of Monte Carlo Simulation for Human Exposure Assessment at a Superfund Site. *Risk Analysis* **1994**, *14*, 433–439, doi:10.1111/j.1539-6924.1994.tb00261.x.
2. MEE *The Chinese Exposure Factors Handbook (Adults）*; China Environmental Science Press: Beijing, 2013; ISBN 978-7-5111-1592-8.
3. Zhang, Y.; Li, C.; Yan, Q.; Han, S.; Zhao, Q.; Yang, L.; Liu, Y.; Zhang, R. Typical Industrial Sector-Based Volatile Organic Compounds Source Profiles and Ozone Formation Potentials in Zhengzhou, China. *Atmospheric Pollution Research* **2020**, *11*, 841–850, doi:10.1016/j.apr.2020.01.012.
4. Wang wenxiu; Wang Yong-min; Zheng Xing-cheng; Zhang Li-na; Zhou Yang; Huang Haoyun Study on VOCs Emission Inventory and Characteristics of Vehicle Maintenance Industry in Tianjin. *Guangzhou Chemical Industry* **2017**, *45*, 123–126.
5. Mo Zi-wei; Lu Si-hua; Li Yue; Shao Min; Qu Hang Emission Characteristics of Volatile Organic Compounds(VOCs) from Typical Solvent Use Factories in Beijing. *China Environmental Science* **2015**, *35*, 374–380.
6. Guha, N.; Loomis, D.; Grosse, Y.; Lauby-Secretan, B.; Ghissassi, F.E.; Bouvard, V.; Benbrahim-Tallaa, L.; Baan, R.; Mattock, H.; Straif, K. Carcinogenicity of Trichloroethylene, Tetrachloroethylene, Some Other Chlorinated Solvents, and Their Metabolites. *The Lancet Oncology* **2012**, *13*, 1192–1193, doi:10.1016/S1470-2045(12)70485-0.
7. Guo, H.; Ding, A.J.; Wang, T.; Simpson, I.J.; Blake, D.R.; Barletta, B.; Meinardi, S.; Rowland, F.S.; Saunders, S.M.; Fu, T.M.; et al. Source Origins, Modeled Profiles, and Apportionments of Halogenated Hydrocarbons in the Greater Pearl River Delta Region, Southern China. *J. Geophys. Res.* **2009**, *114*, 2008JD011448, doi:10.1029/2008JD011448.
8. Chipperfield, M.P.; Hossaini, R.; Montzka, S.A.; Reimann, S.; Sherry, D.; Tegtmeier, S. Renewed and Emerging Concerns over the Production and Emission of Ozone-Depleting Substances. *Nat Rev Earth Environ* **2020**, *1*, 251–263, doi:10.1038/s43017-020-0048-8.
9. Li, B.; Zhao, X.; Li, X.; Hu, X.; Hu, L.; Chen, D.; An, M.; Yang, Y.; Feng, R.; Guo, L.; et al. Emission Factors of Ozone-Depleting Chloromethanes during Production Processes Based on Field Measurements Surrounding a Typical Chloromethane Plant in China. *Journal of Cleaner Production* **2023**, *414*, 137573, doi:10.1016/j.jclepro.2023.137573.
10. Hu, X.; Yao, B.; Fang, X. Anthropogenic Emissions of Ozone-Depleting Substance CH3Cl during 2000–2020 in China. *Environmental Pollution* **2022**, *310*, 119903, doi:10.1016/j.envpol.2022.119903.
11. Liang, X.; Sun, X.; Xu, J.; Ye, D. Improved Emissions Inventory and VOCs Speciation for Industrial OFP Estimation in China. *Science of The Total Environment* **2020**, *745*, 140838, doi:10.1016/j.scitotenv.2020.140838.
12. IARC Working Group on the Evaluation of Carcinogenic Risks to Humans *1,3-Butadiene, Ethylene Oxide, and Vinyl Halides (Vinyl Flouride, Vinyl Chloride, and Vinyl Bromide)*; IARC monographs on the evaluation of carcinogenic risks to humans; International Agency for Research on Cancer: Lyon, France Geneva, 2008; ISBN 978-92-832-1297-3.
13. Hossaini, R.; Sherry, D.; Wang, Z.; Chipperfield, M.; Feng, W.; Oram, D.; Adcock, K.; Montzka, S.; Simpson, I.; Mazzeo, A.; et al. On the Atmospheric Budget of Ethylene

Dichloride and Its Impact on Stratospheric Chlorine and Ozone (2002–2020) 2024.
14. Chen, K.; Gu, X.; Cai, M.; Zhao, W.; Wang, B.; Yang, H.; Liu, X.; Li, X. Emission Characteristics, Environmental Impacts and Health Risk Assessment of Volatile Organic Compounds from the Typical Chemical Industry in China. *Journal of Environmental Sciences* **2025**, *149*, 113–125, doi:10.1016/j.jes.2023.10.023.
15. Cheng, N.; Jing, D.; Zhang, C.; Chen, Z.; Li, W.; Li, S.; Wang, Q. Process-Based VOCs Source Profiles and Contributions to Ozone Formation and Carcinogenic Risk in a Typical Chemical Synthesis Pharmaceutical Industry in China. *Science of The Total Environment* **2021**, *752*, 141899, doi:10.1016/j.scitotenv.2020.141899.
16. Lin, Q.; Gao, Z.; Zhu, W.; Chen, J.; An, T. Underestimated Contribution of Fugitive Emission to VOCs in Pharmaceutical Industry Based on Pollution Characteristics, Odorous Activity and Health Risk Assessment. *Journal of Environmental Sciences* **2023**, *126*, 722–733, doi:10.1016/j.jes.2022.03.005.
17. Zhong, Z.; Ji, Y.; Zhao, M.; Zhou, G.; Hou, Y.; Fan, L.; Ye, D.; Huang, H. Comparison of Emission Characteristics and Risk Assessment of Volatile Organic Compounds of Typical Pharmaceutical Industries in Central Plains, China. *Atmospheric Pollution Research* **2025**, *16*, 102396, doi:10.1016/j.apr.2024.102396.
18. Lv Guoli Studies on the Pollution Characteristics of Volatile Organic Compounds (VOCs) from Pharmaceutical Industry, Hebei University of Science and Technology、, 2013.
19. MEE Emission Standard of Air Pollutants for Pharmaceutical Industry Available online: https://sthjt.zj.gov.cn/art/2022/1/27/art_1201911_58931099.html (accessed on 10 March 2025).
20. The Montreal Protocol on Substances That Deplete the Ozone Layer | Ozone Secretariat Available online: https://ozone.unep.org/treaties/montreal-protocol/montreal-protocol-substances-deplete-ozone-layer (accessed on 13 June 2025).
21. Lin, Y.; Gong, D.; Lv, S.; Ding, Y.; Wu, G.; Wang, H.; Li, Y.; Wang, Y.; Zhou, L.; Wang, B. Observations of High Levels of Ozone-Depleting CFC-11 at a Remote Mountain-Top Site in Southern China. *Environ. Sci. Technol. Lett.* **2019**, *6*, 114–118, doi:10.1021/acs.estlett.9b00022.
22. Fisher, D.A.; Midgley, P.M. The Production and Release to the Atmosphere of CFCs 113, 114 and 115. *Atmospheric Environment. Part A. General Topics* **1993**, *27*, 271–276, doi:10.1016/0960-1686(93)90357-5.
23. Lewis, R.J.; Sax, N.I. Sax's Dangerous Properties of Industrial Materials. Vol. 3: General Chemicals: H - Z. In; Wiley: New York, NY, 2004 ISBN 978-0-471-47662-7.
24. *Toxicological Profile for 1,2-Dibromoethane*; Agency for Toxic Substances and Disease Registry (US): Atlanta (GA), 2018;
25. Ministry of Agriculture of the PRC Announcement No. 199 of the Ministry of Agriculture of the People's Republic of China Available online: https://www.moa.gov.cn/ztzl/ncpzxzz/flfg/200709/t20070919_893058.htm (accessed on 10 March 2025).
26. Wu, J.; Fang, X.; Xu, W.; Wan, D.; Shi, Y.; Su, S.; Hu, J.; Zhang, J. Chlorofluorocarbons, Hydrochlorofluorocarbons, and Hydrofluorocarbons in the Atmosphere of Four Chinese Cities. *Atmospheric Environment* **2013**, *75*, 83–91, doi:10.1016/j.atmosenv.2013.04.031.
27. Lv, Z.; Liu, X.; Wang, G.; Shao, X.; Li, Z.; Nie, L.; Li, G. Sector-Based Volatile Organic

Compounds Emission Characteristics from the Electronics Manufacturing Industry in China. *Atmospheric Pollution Research* **2021**, *12*, 101097, doi:10.1016/j.apr.2021.101097.

28. Xu Zunzhu; Xiong HongHong; Zhang Yuwei; Jin Xiaoxian Emission Characteristics and Emission Reduction Potential of Volatile Organic Compounds from Typical Industrial Sources in Jiangsu Province. *Chinese Journal of Environmental Engineering* **2024**, *18*, 547–559, doi:10.12030/j.cjee.202305040.
29. Xia Bangshou; Zhang Qingchuan; Zhang Shaoxiou; Liu Yue On Emission Characteristics and Prevention Countermeasures of the Electronics Industry Waste VOCs. *Environment and Sustainable Development* **2014**, *39*, 81–83, doi:10.19758/j.cnki.issn1673-288x.2014.05.025.
30. SAC, PRC Limits for Volatile Organic Compounds Content in Cleaning Agents Available online: https://openstd.samr.gov.cn/bzgk/gb/newGbInfo?hcno=FE1FC015A8AC8E87F74085C3ADE06C3E (accessed on 13 June 2025).
31. PUBLIC HEALTH STATEMENT FOR 1,1-DICHLOROETHANE. In *TOXICOLOGICAL PROFILE FOR 1,1-DICHLOROETHANE*; Agency for Toxic Substances and Disease Registry (US), 2015.
32. Zuo, H.; Jiang, Y.; Yuan, J.; Wang, Z.; Zhang, P.; Guo, C.; Wang, Z.; Chen, Y.; Wen, Q.; Wei, Y.; et al. Pollution Characteristics and Source Differences of VOCs before and after COVID-19 in Beijing. *Science of The Total Environment* **2024**, *907*, 167694, doi:10.1016/j.scitotenv.2023.167694.
